# Global disruption of degree rank order: a hallmark of chronic pain

**DOI:** 10.1038/srep34853

**Published:** 2016-10-11

**Authors:** Ali Mansour, Alex T. Baria, Pascal Tetreault, Etienne Vachon-Presseau, Pei-Ching Chang, Lejian Huang, A. Vania Apkarian, Marwan N. Baliki

**Affiliations:** 1 Department of Physiology, Northwestern University, Feinberg School of Medicine, Chicago, Illinois 60610, USA; 2 Department of Physical Medicine and Rehabilitation, Northwestern University, Chicago, Illinois 60611, USA; 3 Rehabilitation Institute of Chicago, Chicago, Illinois 60611, USA

## Abstract

Chronic pain remains poorly understood; yet it is associated with the reorganization of the nervous system. Here, we demonstrate that a unitary global measure of functional connectivity, defined as the extent of degree rank order disruption, *k*_*D*_, identifies the chronic pain state. In contrast, local degree disruption differentiates between chronic pain conditions. We used resting-state functional MRI data to analyze the brain connectome at varying scales and densities. In three chronic pain conditions, we observe disrupted *k*_*D*_, in proportion to individuals’ pain intensity, and associated with community membership disruption. Additionally, we observe regional degree changes, some of which were unique to each type of chronic pain. Subjects with recent onset of back pain exhibited emergence of *k*_*D*_ only when the pain became chronic. Similarly, in neuropathic rats *k*_*D*_ emerged weeks after injury, in proportion to pain-like behavior. Thus, we found comprehensive cross-species evidence for chronic pain being a state of global randomization of functional connectivity.

After more than a century of studying nociception and its underlying pathways, applicability of this information to chronic pain remains unclear. Chronic pain is highly rampant, with a staggering health care cost, and it has become the leading cause of years lived with disability in the USA and the seventh leading cause worldwide^1^. Evidence derived from rodent models of chronic/persistent pain attest to the reorganization of the nervous system, mostly emphasizing changes in afferent nociceptors and spinal cord circuitry^2^,^3^. Advances in human brain imaging technology over the past decades have enabled direct probing of brain properties in clinical chronic pain conditions. Studies from various patient cohorts now show that chronic pain is not just correlated to nociceptive processes, but also associated with large-scale brain functional and morphological reorganization^4^,^5^,^6^. In addition to encoding the clinical pathology (e.g. pain intensity and duration), brain properties play an integral part in the development of chronic pain after an inciting injury^7^,^8^. These observations allude to the brain playing a critical role in chronic pain. Still, it remains unknown whether chronic pain is an identifiable specific brain state.

Human brains have topological attributes in common with other complex systems that are thought to provide the physiological basis for information processing^9^,^10^ and to reflect the cumulative effect of learned experiences throughout a lifetime^11^. Multiple chronic pain conditions are associated with aberrant resting state properties^12^,^13^,^14^,^15^,^16^,^17^ impacting brain regions critical for learning^18^. Therefore, we hypothesized that chronic pain may be characterized as an abnormal brain network state, which could reflect the clinical properties of the condition, and even allow us to identify the time window within which acute pain transitions into chronic pain.

To determine the specificity (or ubiquity) of brain functional changes in the presence of chronic pain, we first examined the impact of chronic pain on the topology of brain networks in individuals suffering from different chronic pain conditions including prolonged chronic back pain (CBP), complex pain regional syndrome (CRPS) and osteoarthritis (OA). We also, over a one-year observation period, followed subjects with an episode of sub-acute back pain (SBP) as they transitioned to chronic pain, in order to determine the temporal properties of functional reorganization underlying the emergence of chronic pain. We validated our primary observations in two novel groups of CBP and OA patients. Finally, for cross-species generalization, we performed a similar investigation of the transition to a persistent neuropathic pain condition in a rodent model.

We used resting state functional MRI data to construct brain graphs of regional cortical and subcortical nodes, with edges (or links) drawn between nodes representing their functional correlation. Brain networks were constructed and studied at different parcellations and correlation thresholds, resulting in sparse but fully connected networks. We compared global and local nodal brain network properties between patients, and age- and gender-matched healthy controls. To ensure that outcomes were generalizable to chronic pain cohorts at large, all global connectivity comparisons were performed relative to an offsite control data set. A similar analysis was also applied to rat resting state fMRI collected under isoflurane anesthesia. In both species, we asked whether functional connectivity reorganization is dependent on time from onset of symptoms, and whether the extent of disruption is related to the pain, its clinical correlates, or to pain-like behavior in the rat.

## Results

### Chronic pain does not alter global network topological properties

**Figure 1:**
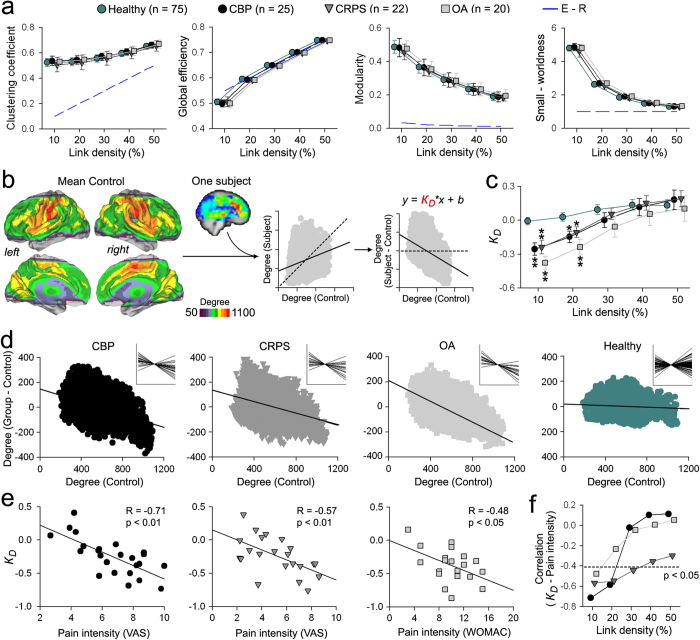
The brain in chronic pain exhibits disrupted degree rank order in proportion to the intensity of chronic pain. (**a**) Global graph properties of resting state fMRI brain networks do not differentiate between chronic pain patients and healthy controls. Clustering (group effect: F_(3,136)_ = 1.88, p = 0.14), global efficiency (F_(3,136)_ = 1.70, p = 0.17), modularity (F_(3,136)_ = 2.18, p = 0.10) and small–worldness (F_(3,136)_ = 2.23, p = 0.09) were similar for all groups (blue lines represent data from equivalent Erdos – Renyi, random graphs). Data plotted as mean ± s.e.m. **(b)** Method for deriving *k*_*D*_: Cortical surface representation of the mean degree map from off-site healthy controls (n = 129, at 10% link density, Supplementary Fig. 1). For any given subject, the mean degree of each node in the off-site healthy control group (x axis) is first plotted versus degree value for that node in the subject. Instead for each node degree value, if we plot the difference in degree for that node (subject – off-site control) across all nodes (y axis), then the slope of the fitted data measures *k*_*D*_. **(c)** Mean ± s.e.m. of *k*_*D*_ in our healthy and pain patients across all link densities. Patient groups exhibited significant degree rank order disruption compared to off-site healthy controls (group effect: F_(3,136)_ = 12.56, p < 0.001). Differences were only observed for link densities < = 20% (*p < 0.05; **p < 0.01, Tukey post-hoc compared to healthy). (**d**) Scatter plots depict the group *k*_*D*_ for CBP, CRPS, OA and our healthy subjects compared to the off-site healthy control group for 10% link density. Inserts show individual *k*_*D*_ values for each group. (**e**) *k*_*D*_ showed a significant relationship to pain intensity in CBP (R = −0.71, p < 0.001), CRPS (R = −0.57, p = 0.006) and OA (R = −0.48, p = 0.03), at 10% link density. (**f**) Correlation between *k*_*D*_ and pain intensity across all link density densities. *k*_*D*_ was significantly related to pain intensity only when the brain networks were in the small-world regime (dashed line represents p = 0.05 for df = 20).

Twenty-five CBP patients (Supplementary Table 1), 22 CRPS patients (Supplementary Table 2), 20 OA patients (Supplementary Table 3), and 75 healthy controls, recruited from Chicago and imaged on our 3 Tesla Siemens Trio scanner, participated in this part of the study. We constructed brain graphs of 5,828 regional cortical and subcortical nodes (216 = 6 × 6 × 6 mm^3^ isometric voxels), with edges (or links; 16,979,878 total possible) drawn between nodes to represent their functional correlation. Network measures are greatly influenced by basic network characteristics, such as the number of nodes and distribution links^19^. Because there is no “correct” threshold at which to analyze a graph^20^, brain networks were constructed and studied at different correlation thresholds. This resulted in sparse but fully connected networks (10% of total possible links) and in more densely connected networks (50%). In order to better understand the effect of thresholding on brain networks, we performed the same analysis on a null-hypothesis network that has a random topology but shares the size, density and number of connections of the brain networks. Overall, there were no significant differences between groups on global measures of network topology. Functional networks in all groups had similar global efficiency (a measure of information integration), clustering (a measure of information segregation) and modularity (a measure of the near-decomposability of the network into a community structure of sparsely interconnected modules) across all link densities examined (repeated measure ANCOVA with age and gender as covariates of no interest) (Fig. 1a). Thus, despite the marked differences in clinical state between patients and healthy subjects, brain networks showed similar global properties between patients and healthy subjects.

It is important to note that graph properties for healthy subjects and patients diverged from those of random graphs for densities ≤20%. For example, all groups demonstrated the characteristic small-world property^21^ of high clustering, combined with high global efficiency (normalized to clustering and efficiency of random networks with the same number of nodes, connection density and degree distribution) for link densities ≤20% (Fig. 1a). Given that graph theoretic techniques are most meaningful in sparse graphs (Newman, 2010), we focus our analysis on brain graphs constructed at 10% link density since this produced the most viable brain graphs, with similar global properties to those reported in previous studies^9^,^19^,^22^.

### Chronic pain is associated with whole-brain degree rank order disruption that reflects clinical properties

An earlier study shows that, in the absence of global topological changes, subjects may exhibit large disturbances in functional connectivity^23^. We therefore tested whether the brain in chronic pain exhibits whole-brain reorganization of functional connectivity. We used a unitary measure which assesses whole-brain degree rank order disruption (*k*_*D*_), defined as the gradient fitted to the mean difference in nodal degree between any given subject (or group of subjects) in relation to the mean nodal degree in a control population across all nodes^23^. We assessed *k*_*D*_ for all of our patients and our healthy subjects in relation to an off-site age- and gender-matched healthy control dataset (n = 129, Fig. 1b, Supplementary Fig. 1, Supplementary Table 4) taken from Connectome1000^24^. Overall, all patient groups (but not our healthy group) showed significant degree rank order disruption (i.e. negative *k*_*D*_ values) compared to the off-site controls, for link densities ≤20% (Fig. 1c, Supplementary Table 5). In other words, nodes that had high degree scores in off-site healthy controls (highly connected) showed reductions in counts in patients, whereas the nodes that had low degree scores in off-site controls showed increased counts in the patients. Furthermore, this nodal reorganization was observed only when the networks exhibited small world properties. Moreover, we found that this disrupted connectivity profile was consistently demonstrated for the group-averaged degree maps, as well as for the majority of the individual patients’ degree maps (Fig. 1d).

Pain intensity is a fundamental clinical parameter characterizing chronic pain, and a primary reason for patients to seek health care. We observed that subjectively-reported intensity of pain at the time of brain scan showed a strong negative correlation with individual *k*_*D*_ scores in all patient groups, for 10% link density (Fig. 1e). Furthermore, for link densities ranging from 10–50%, this relationship between *k*_*D*_ and pain was only observed when networks exhibited small world properties (Fig. 1f).

Previous studies have shown that network properties of resting state networks vary depending on the parcellation resolution^10^. Here, we sought to investigate how such variations in parcellation templates affect *k*_*D*_. We reproduced our results using lower resolution templates with 480 and 90 nodes (Supplementary Fig. 2). We also assessed the resilience of the *k*_*D*_measurement on the level of noise contamination in the BOLD time series. We found that our patients and our healthy subjects showed consistent *k*_*D*_ values relative to off-site controls, even if 40% of the original BOLD signal was replaced by white Gaussian noise (Supplementary Fig. 3).

Taken together, these results show that chronic pain is associated with a robust change in *k*_*D*_ in multiple types of chronic pain when the networks exhibit small world properties. Moreover, this disruption in functional connectivity reflects individual subjective pain intensity, is resilient to the addition of white noise, and can be observed at different parcellation scales.

### Chronic pain is associated with disrupted community structure

**Figure 2:**
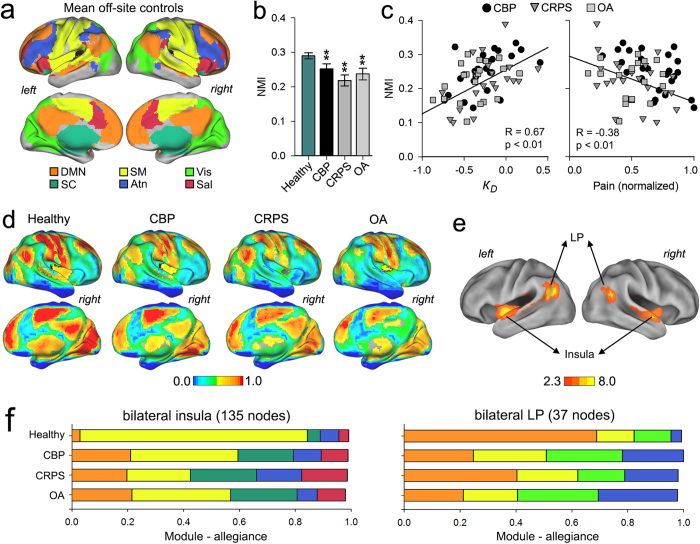
The brain in chronic pain exhibits disruption of community membership. **(a)** Cortical surface representation of the group mean community structure for off-site healthy controls, different colors represent different modules. **(b)** Mean ± s.e.m normalized mutual information (NMI) for all groups measured with respect to the mean off-site healthy control. NMI showed a significant group effect (F_(3,136)_ = 12.40, p < 0.001), with CBP (0.24 ± 0.01), CRPS (0.21 ± 0.01) and OA (0.23 ± 0.01) exhibiting lower NMI compared to the healthy (0.29 ± 0.01) (*p < 0.05; **p < 0.01, Tukey post - hoc compared to healthy). **(c)** NMI of all patients with respect to the mean off-site control showed a significant correlation to *k*_*D*_ (r = 0.67, p < 0.001) and to pain intensity (r = −0.38, p = 0.006). (**d**) Cortical surface representation of the group mean module-allegiance indicating the probability of a given node will be located in the same functional community of off-site healthy controls. Overall, nodes located at the edges of communities showed the lowest module-allegiance across all groups. (**e)** Location of nodes with significant module-allegiance differences across groups (f–zscore > 2.3, p < 0.01, FEW corrected). Nodes showing differences in module-allegiance were localized to bilateral insula (135 nodes within SM module) and lateral parietal cortex (LP, 37 nodes within the DMN module). (**f**) Bar graphs show the group average module-allegiance for the insula (left) and LP (right) to all communities in patients and healthy subjects. The insular nodes showed decreased module-allegiance with SM module (F_(3,136)_ = 9.11, p < 0.001) and increased module-allegiance with the DMN (F_(3,136)_ = 8.74, p < 0.001) and SC module (F_(3,136)_ = 7.04, p < 0.001) in patients compared to healthy subjects. The LP nodes showed decreased module-allegiance with DMN module (F_(3,136)_ = 11.34, p < 0.001) and increased module-allegiance with the Atn (F_(3,136)_ = 7.75, p < 0.001) in patients compared to healthy subjects. (ANCOVA with age and gender as covariates of no interest, corrected for multiple comparisons using Bonferroni).

The *k*_*D*_ metric provides a global measure of the extent of functional connectivity disruption, which may be the consequence of a multiplicity of reorganizational principles. To unravel mechanisms that may underlie observed *k*_*D*_ changes in chronic pain patients, we examine these brain networks at an intermediate scale of organization, which is better described by the community structure or modularity of the network^25^,^26^. Modularity is a measure of how well a network can be dissected into a set of sparsely interconnected communities or modules, where nodes within a module are densely intra-connected. Here we investigated possible changes in modular architecture associated with chronic pain and its relationship to *k*_*D*_. First, we generated a baseline community structure from the off-site control data, closely following methods recently described^20^. Consistent with previous reports^27^,^28^, the off-site control resting state functional connectivity map was segregated into 6 modules corresponding to commonly observed large-scale resting state networks, including the default-mode (DMN), salience (Sal), sensorimotor (SM), attention (Atn), visual (Vis), and subcortical (SC) networks (Fig. 2a). We then used normalized mutual information (NMI) to quantify the overall similarity of community structure of all our patients and healthy subjects in relation to the off-site control community. Although the number of functional modules was similar between our healthy controls and patients (see Fig. 1a), the community structure (node membership) was perturbed in the patients. All of our patient groups showed significantly lower NMI than our healthy volunteers compared to the off-site healthy group (Fig. 2b). Furthermore, the individual subject NMI values were positively correlated to *k*_*D*_ and negatively correlated to pain intensity (Fig. 2c).

Next, we sought to localize the nodes exhibiting differences in their modularity membership in chronic pain patients. For each subject (healthy or patient), we examined the nodal module-allegiance with respect to the baseline community structure of the off-site control group data. Nodes that showed the same modular membership to controls were assigned a value of 1, whereas nodes exhibiting a different membership were assigned a value of 0. Group average module-allegiance maps for the healthy and patient groups displayed several interesting anatomical features, including nodes that were consistently grouped into the same community. Others – mainly located at the edges of communities – did not show consistent allegiances to any functional module (Fig. 2d).

Statistical differences in nodal module-allegiance between groups were assessed using a whole-brain ANCOVA. Of all nodes examined, a cluster of 135 nodes within the bilateral insula (members of the SM module) and a cluster of 35 nodes with the bilateral lateral parietal (LP) cortices (members of the DMN) showed significant differences in module-allegiance across groups (Fig. 2e). We observed that these nodes show a split allegiance in patients compared to healthy subjects. The insular nodes showed decreased allegiance to the SM module in CBP (38.58%), CRPS (22.73%) and OA (35.33%) compared to healthy subjects (79.92%). This was accompanied by increases in module-allegiance to both the DMN and SC module in CBP (20.94% and 19.79%), CRPS (19.72% and 23.67%), OA (21.52% and 23.70%) compared to healthy subjects (4.42% and 4.58% respectively). The subset of nodes within the LP cortices showed decreased allegiance to the DMN and increased allegiance to the Atn module in CBP (24.67% and 21.78%), CRPS (40.33% and 19.05%) and OA (21.11% and 28.36%) compared to healthy subjects (68.81% and 3.01%, respectively) (Fig. 2f). The insula and LP allegiance changes did not show dependence on type of chronic pain.

Lastly, we investigated the contribution of the localized nodal module-allegiance changes within the insular and lateral parietal regions to the overall modular changes observed in patients compared to healthy subjects (see Fig. 2b). We used NMI to recalculate the overall similarity of community structure of all our patients and healthy subjects after removing the 135 insular and 35 lateral parietal nodes that exhibited significant differences in module-allegiance in patients. We observed that, even after discounting the effect of the localized changes, patient groups showed significantly lower NMI than our healthy volunteers compared to the off-site healthy group. In addition, the individual subject NMI values were still positively correlated to *k*_*D*_ and negatively correlated to pain intensity (Supplementary Fig. 4). These results demonstrate that the extent of the overall modularity disruption (~20% from controls) is not driven by the insular and parietal regions, since these showed similar patterns of module-allegiance changes across types of chronic pain.

### Anatomical localization of nodal degree changes

**Figure 3:**
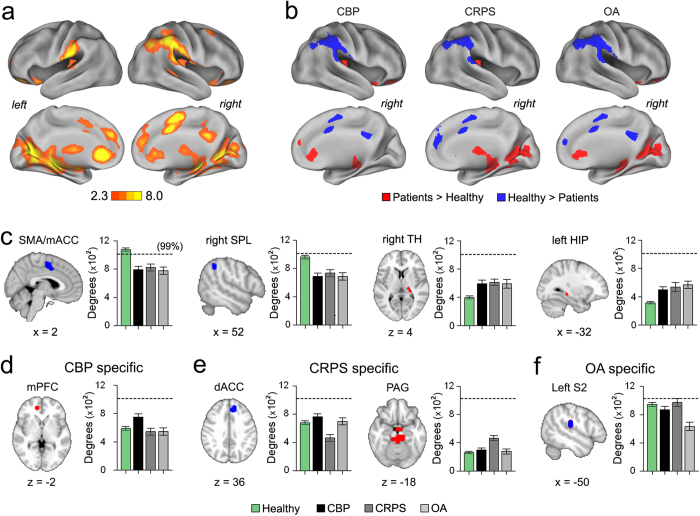
Chronic pain conditions have common and specific local nodal degree changes. **(a)** Cortical surface maps for difference in nodal degree across all groups for 10% link density analysis (whole-brain voxelwise ANCOVA with age and gender as covariates of no interest, f – zscore > 2.3, p < 0.01, FWE corrected using threshold – free cluster enhancement). **(b)** Cortical surface maps illustrate nodes that show differences between patients and our healthy controls for 10% link density analysis (Tukey post - hoc compared to healthy, 0070 < 0.05 FDR corrected). Red denotes significantly increased degree and blue denotes significantly decreased degree in CBP, CRPS and OA compared to healthy. (**c**) Brain slices show regions that exhibited common decreases (blue) and increases (red) in nodal degree in all patients compared to our healthy subjects (conjunction of statistical maps in Fig. 2b). Brain regions that showed decreased connections included SMA/mACC and right SPL and brain regions that showed significant increases included right TH and left HIP. Bar graphs show the corresponding mean ± s.e.m of nodal degree. (**d**–**f**) Brain regions that showed patient specific nodal degree changes for CBP, CRPS and OA compared to all other patient groups and healthy subjects (regions were determined using a conjunction analysis of relevant post-hoc comparisons, see Supplementary Fig. 5). CBP patients showed increased nodal degree in mPFC CRPS showed decreased nodal degree in dACC and increased nodal degree in PAG; OA patients exhibited decreased nodal degree in left S2. Bar graphs show the corresponding mean ± s.e.m of nodal degree. Dashed line represents the 99 percentile for degree counts in our healthy group. Coordinates and cluster sizes for all regions that showed nodal degree changes are listed in Supplementary Table 6.

Classically, brain disturbances in relation to pain have been described as localized changes in the functioning of individual brain regions, or in the altered connectivity strength of specific pathways. Similarly, here we investigated local (nodal or voxel-based) degree disturbances in our patients, expecting to discover the relationship between local and whole-brain reorganization. Overall, 755 nodes showed significant degree changes across all groups and were localized to a distributed set of regions including the medial prefrontal cortex (mPFC), nucleus accumbens (NAc), supplementary motor area extending into the mid-anterior cingulate cortex (SMA/mACC), precuneus, superior parietal lobe (SPL), thalamus (TH), hippocampus (HIP), secondary somatosensory cortices (S2), periaqueductal gray (PAG) and visual cortex (Fig. 3a, Supplementary Fig. 5).

Significant increases and decreases in nodal degree between any two groups (healthy or chronic pain) were determined using post-hoc analysis (Supplementary Fig. 5). Compared to healthy subjects, (1) CBP patients showed abnormally decreased connectivity in SMA/mACC, precuneus and SPL, and increased connectivity in the right posterior TH, left HIP, bilateral NAc and mPFC. (2) CRPS patients showed decreased nodal degree in SMA/mACC, SPL, fronto-parietal areas and dACC, and increased connectivity primarily in bilateral TH and HIP, PAG and visual regions. (3) OA patients showed decreased nodal degree in very similar areas as CBP, and increased nodal degree in areas observed to CBP and CRPS (Fig. 3b). Nodes that showed common degree changes across the three patient groups were determined using a conjunction analysis. Overall, brain regions that showed decreased connectivity for all three patient groups compared to our healthy subjects included the SMA/mACC, part of the somatosensory network, and the right SPL, a region within the attention network (Fig. 3c, Supplementary Fig. 6a, Supplementary Table 6). On the other hand, two regions showed similar increased connectivity for all patient groups compared to healthy subjects and were localized to the right posterior TH and left HIP (Fig. 3c, Supplementary Fig. 6b, Supplementary Table 6).

We also identified brain regions that showed specific patient type-dependent nodal degree changes for CBP, CRPS and OA. Nodes were considered to be patient type-specific only if they exhibited regional significant decreases or increases compared to all other patient groups and healthy subjects (Supplementary Fig. 5). CBP was associated with specific increased connectivity in the mPFC part of the DMN (Fig. 3d, Supplementary Fig. 6c, Supplementary Table 6). CRPS was associated with specific increased connectivity in the PAG and decreased connectivity within the dACC (Fig. 3e, Supplementary Fig. 6d, Supplementary Table 6), whereas OA only showed specific decreased nodal degree in the left S2, part of the somatosensory network (Fig. 3f, Supplementary Fig. 6e, Supplementary Table 6). Finally, we investigated the reproducibility of our results at different link densities. Overall, all brain regions investigated (either commonly or specifically perturbed in the patient groups) showed consistent changes in nodal degree across different link densities, especially for link densities ≤30% (Supplementary Fig. 7).

Overall, we observe local functional connectivity changes, some regions specific to each condition, and others observed commonly across types of chronic pain. We next examine the interrelationships between these local changes and the larger scale perturbations.

### Degree rank order disruption permeates the whole-brain network

**Figure 4:**
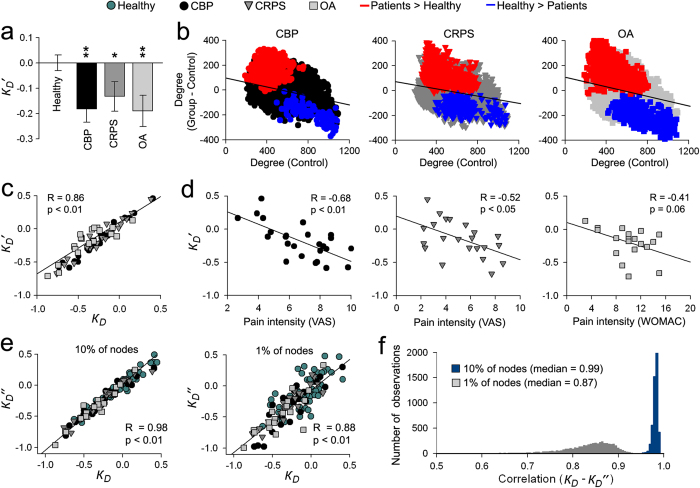
Degree rank order disruption in chronic pain reflects globally altered connectivity. (**a**) Bar graph shows the mean ± s.e.m of the adjusted degree rank order disruption (*k*_*D*_’) that was computed after deleting all nodes that showed significant differences between patients and off-site healthy subjects, from Fig. 3a for 10% link density analysis. Patients exhibited significant degree rank order disruption compared to healthy controls (group effect: F_(3,136)_ = 4.51, p = 0.005, ANCOVA with age and gender as covariates of no interest), with CBP (−0.18 ± 0.06), CRPS (−0.13 ± 0.06) and OA (−0.19 ± 0.06) showing significantly lower *k*_*D*_*’* compared to our healthy controls (0.00 ± 0.03; *p < 0.05; **p < 0.01, Tukey post - hoc). (**b**) Scatter plots depict group *k*_*D*_’ for CBP, CRPS, OA and our healthy controls, as compared to the off-site control group for 10% link density analysis. Blue and red points denote the deleted nodes that showed significantly decreased or increased degree compared to the off-site healthy control group. **(c)** *k*_*D*_ and *k*_*D*_*’* exhibited a strong association across all patients (R = 0.98, p < 0.001). **(d)** *k*_*D*_*’* showed a significant relationship with pain intensity in CBP (R = −0.68, p < 0.001), CRPS (R = −0.52, p = 0.02), and a trend in OA (R = −0.41, p = 0.06). **(e)** Scatter plots show the relationship between *k*_*D*_ and the rank order disruption (*k*_*D*_*”)* that was computed from 10% (left plot) or 1% (right) of randomly selected nodes. *k*_*D*_ was strongly correlated to *k*_*D*_*”* estimated for 10% of nodes (R = 0.98, p < 0.001) and for 1% of nodes (R = 0.88, p < 0.001). **(f)** Graph shows the distributions of correlation of *k*_*D*_with *k*_*D”*_ computed for 10% (blue, median = 0.99) and 1% (gray, median = 0.88) of nodes, over 5000 permutations.

In addition to a perturbed *k*_*D*_ and an abnormally variable community structure, patient groups exhibited consistent (and statistically highly significant) spatial patterns of brain regions gaining and losing functional connectivity. Here, we investigated the contribution of these regional perturbations to *k*_*D*_. Individual *k*_*D*_ values were recomputed in relation to the off-site healthy group after deletion of all nodes showing significant group differences in degree (increases or decreases) compared to our healthy subjects (denoted as *k*_*D’*_). This deletion was not sufficient to renormalize individual subject rank order disruption. *k*_*D’*_ was significantly lower in all patient groups compared to healthy subjects at 10% link density, showed high correlation with *k*_*D*_, and was significantly correlated with pain intensity for all patient groups (Fig. 4a–d).

We further explored the effect of nodal specificity on degree rank order disruption index by computing *k*_*D*_from a random 10% or 1% subset of nodes in patients and healthy subjects (denoted as *k*_*D”*_). Surprisingly, *k*_*D”*_ showed similar values to *k*_*D*_ (Fig. 4e), that was consistent across 5000 different random permutations of nodes (Fig. 4f). These results imply that the degree rank order disruption observed in chronic pain patients reflects altered connectivity properties permeating all of the brain and is not driven by functional changes to specific brain regions or pathways.

### Multi–factor regression analysis identifies *k*_*D*_as the primary parameter related to chronic pain intensity

We identified multiple interrelated brain network properties that are altered in chronic pain patients. Here, we explore their interaction and relation to predicting intensity of chronic pain using a multi-factorial regression analysis. We first investigated dependence of chronic pain intensity on clinical (pain duration, depression) and demographic (gender and age) parameters, in addition to localized (nodal degree of SMA/mACC, right SPL, right TH, left HIP, mPFC, dACC, PAG and left S2) and global brain network parameters including *k*_*D*_, modularity, clustering, and efficiency. Pain intensity showed independent strong associations with *k*_*D*,_ NMI, and localized nodal degree, with *k*_*D*_ showing the highest correlation with pain intensity (Supplementary Table 7). We also investigated the dependence of chronic pain intensity on the various parameters using a backward and forward stepwise multiple regression analysis. In both analyses, intensity of chronic pain showed significant association only with *k*_*D*_ (Supplementary Table 8).

### Subacute back pain patients exhibit emergence of increased degree rank order disruption with transition to chronic pain

**Figure 5:**
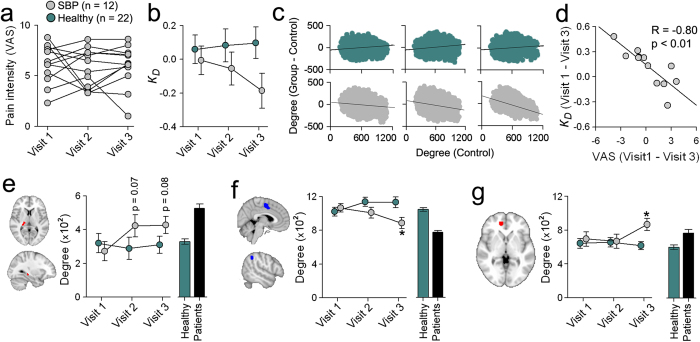
Transition from subacute to chronic back pain is associated with increasing degree rank order disruption. **(a)** Pain ratings in 12 subacute back pain subjects (SBP), studied over 1 year. SBP rank order disruption was examined at 10 weeks, 6 months, and 12 months (visits 1, 2, 3) after onset of symptoms. On average, SBP patients showed no change in reported back pain intensity across the 3 visits (F_(2,22)_ = 0.23, p = 0.80, repeated measure ANOVA). (**b**) Mean ± s.e.m for *k*_*D*_ in SBP (gray circles) and matched healthy controls (green circles, n = 12) for the three visits across all scans, for 10% link density relative to off-site controls. SBP patients showed decreased degree rank order disruption in time compared to the matched healthy controls (group by time effect: F_(2,40)_ = 2.11, p = 0.09, repeated measure ANCOVA with age and gender as covariates of no interest). **(c)** Scatter plots depict the group *k*_*D*_ for the matched healthy controls and SBP patients across all three visits. (**d**) Within-subject changes in *k*_*D*_ showed a significant relationship with corresponding changes in back pain intensity between visits 1 and 3 (R = −0.80, p = 0.007). (**e**) SBP patients showed increased connectivity in hippocampus and thalamus (regions that showed increased nodal degree in chronic pain patients, Fig. 3b) in time compared to healthy controls (group by time effect: F_(2,40)_ = 3.35, p = 0.04, repeated measure ANCOVA with age and gender as covariates of no interest). Bar graphs represent the mean ± s.e.m degree of all our healthy controls and all patients from Fig. 3. (**f**) SBP patients showed decreased connectivity in regions that showed decreased nodal degree in chronic pain patients) in time compared to healthy controls (group by time effect: F_(2,40)_ = 3.13, p = 0.05, repeated measure ANCOVA with age and gender as covariates of no interest). Bar graphs represent the mean ± s.e.m degree of all our healthy controls and all patients from Fig. 3.

Thus far, it remains unclear whether the relationship we have uncovered in the patients between *k*_*D*_ and pain intensity simply reflects the presence of pain, or if it is specific to the chronic pain state. We tackle this issue by examining longitudinal changes in the brain functional properties in 12 SBP patients (Supplementary Table 9) as they transitioned into chronic pain state following an acute inciting event. Resting state fMRI was collected at entry into the study (about 11 weeks after symptom onset, visit 1), and at 6 (visit 2) and 12 (visit 3) months from onset of symptoms. SBP patients showed no change in reported back pain intensity (F_(2,22)_ = 0.44, p = 0.65, repeated measure ANOVA) and depression (BDI, F_(2,22)_ = 0.49, p = 0.61, repeated measure ANOVA) across the 3 visits. Back pain intensity was high (around 6/10) and constant across all visits, thus all 12 SBP patients were transitioning to chronic pain (Fig. 5a). Brain functional networks of these SBP patients showed increased degree rank order disruption in time, in comparison to a sub-set of our healthy controls matched for age and gender, where both (SBP and healthy subset) groups were contrasted to the off-site healthy control (Fig. 5b,c). Even though intensity of back pain on average was constant throughout the year, changes in *k*_*D*_ showed a significant relationship with corresponding changes in back pain intensity between visits 1 and 3 (Fig. 5d), but not visits 1 and 2 (R = −0.07, p = 0.84), suggesting that changes in *k*_*D*_ from baseline reflect back pain intensity only when the pain persists for about one year.

In addition to changes in *k*_*D*_, we investigated localized degree changes in brain regions that showed significant differences for all three of our patient groups. Both the TH and HIP regions showed significant increased connectivity in time in SBP patients, compared to healthy subjects that started to diverge at visit 2 (i.e. 6 months after injury) (Fig. 5e). On the other hand, SPL and SMA/mACC regions showed decreased connectivity only at visit 3 compared to healthy subjects (Fig. 5f). Similarly, the mPFC, which showed increased connectivity in CBP patients, showed increased connectivity in SBP ones only at visit 3 (i.e. when SBP patients’ pain was becoming chronic), compared to matched healthy subjects (Fig. 5g). Therefore, these results imply that the relationship between *k*_*D*_ and back pain intensity is an emergent property observed at a time when the network has stabilized into a more chronic pain-like state.

We also examined degree rank order disruption in a subgroup of CBP patients (n = 12) and healthy subjects (n = 12), matched for gender and age, during an acute thermal pain paradigm, from a previously published data set^29^. CBP patients did not show any significant degree rank order disruption compared to healthy controls for acute thermal pain (Supplementary Fig. 8), demonstrating that brain functional networks underlying acute pain processing are equivalent between patients and healthy subjects, and do not account for presence of degree rank order disruption in CBP.

### Degree rank order disruption in chronic pain patients shows temporal reliability

The rank order disruption we observe in patients was based on a cross-sectional contrast. Here, we perform a longitudinal analysis to examine changes in rank order disruption in a subgroup of CBP patients (n = 9, pain duration mean ± s.e.m. = 14.41 ± 2.55 years) compared to healthy controls (n = 9) over a six-month period, with *k*_*D*_ measured relative to off-site controls. On average, CBP patients showed comparable *k*_*D*_ on both visits compared to those observed in Fig. 1. Similar to our initial observation, *k*_*D*_ in CBP exhibited a strong correlation to back pain intensity at visit 1 and visit 2. Furthermore, changes in individual *k*_*D*_ values between visit 2 and visit 1 were inversely proportional to changes in reported pain intensity. These results corroborate the reliability and ability of *k*_*D*_ to track individual variations in chronic pain intensity, and demonstrate its clinical relevance (Supplementary Fig. 9).

### Rank order disruption predicts pain intensity in two novel groups of CBP and OA patients

*k*_*D*_ showed significant correlation with pain intensity across all three chronic patient groups and in SBP patients one year after the onset of injury, implying its tight coupling with chronic pain intensity. Therefore, we tested whether pain intensity can be estimated directly from *k*_*D*_, using 2 novel groups of CBP (n = 15, Supplementary Table 10) and OA (n = 20, Supplementary Table 11) patients relative to off-site healthy controls. In both patient groups, we observed a significant correlation between observed pain values and pain values derived from individual subject estimates of *k*_*D*_ (Supplementary Fig. 10). We also investigated whether patients from the novel groups can be accurately classified (OA or CBP) based on nodal degree of brain regions exhibiting condition-specific disruptions – the mPFC for CBP and left S2 for OA. We found that while nodal degree in the mPFC significantly differentiated CBP from OA patients, nodal degree of the left S2 did not distinguish OA patients from CBP ones (Supplementary Fig. 10). These results show that global brain functional network reorganization can accurately predict pain intensity, and only some regional changes in connectivity distinguish between types of clinical conditions.

### Animal model of chronic neuropathic pain shows time-dependent rank order disruption, reflecting pain-like behavior

**Figure 6:**
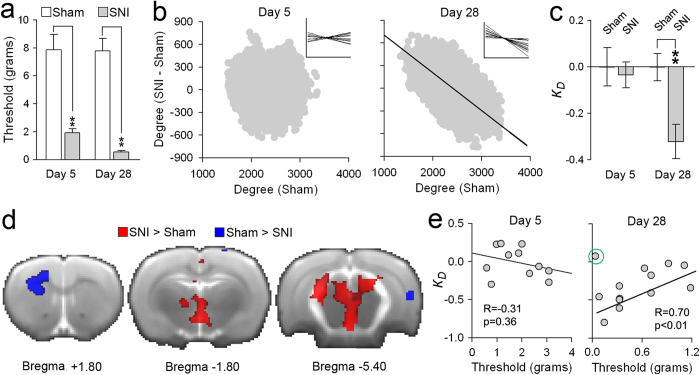
Degree rank order disruption of brain functional networks in rats with spared nerve injury (SNI). (**a**) Mean ± s.e.m. for tactile sensitivity threshold of the injured paw in two groups of SNI and sham animals, day 5 (sham: n = 12, SNI: n = 11) and day 28 (sham: n = 11, SNI: n = 13) post-injury. Both SNI groups exhibited decreased mechanical thresholds (tactile allodynia, pain-like behavior) compared to sham animals, day 5 (sham: 7.89 ± 1.21 grams; SNI: 1.91 ± 0.03 grams; t_(21)_ = −5.21, p = 0.005) and day 28 (sham: 7.78 ± 1.07 grams; SNI = 0.62 ± 0.01 grams; t_(22)_ = −9.19, p < 0.001) (**p < 0.01, two-sided unpaired t-test). **(b)** Scatter plots depict the group *k*_*D*_ of resting state functional networks in SNI animals for 10% link density, in comparison to time-matched sham animals degree counts. Inserts show *k*_*D*_ computed for each animal. **(c)** Mean ± s.e.m for *k*_*D*_ in SNI animals, compared to sham at day 5 and day 28. SNI showed a significant degree rank disruption at day 28 (mean ± s.e.m. = −0.32 ± 0.07, t(22) = −3.40, p = 0.004), but not at day 5 (mean ± s.e.m. = −0.01 ±0.1, t_(21)_ = −0.11, p = 0.91) (**p < 0.01, two-sided unpaired t-test). **(d)** Group differences in degree between SNI and sham at day 28. Areas depicted in red represent increased degree in SNI, and include PAG, ventral tegmentum, hypothalamus, thalamus, and cingulate and NAc shell. Regions in blue show decreased degree in SNI, and include primary sensorimotor, caudate and hippocampus (whole brain contrast p < 0.05 corrected for multiple comparisons using FWE). There were no differences in degree at day 5, relative to day 5 sham. **(e)** Correlations between *k*_*D*_ and tactile sensitivity in SNI animals, at days 5 and 28. SNI animals showed a significant correlation between *k*_*D*_ and tactile sensitivity at day 28 (R = 0.70, p = 0.009, data point delineated by green circle was excluded from the regression), but not at day 5 (R = −0.31, p = 0.36).

To establish cross-species generalizability of our findings, we examined brain functional network rank order reorganization in a rat neuropathic pain model. Resting state fMRI was collected in rats under isoflurane anesthesia at 5 or 28 days following peripheral spared nerve (SNI)^30^ or sham injury^31^. The SNI animals showed a robust increase in touch sensitivity (tactile allodynia, decrease in stimulus response threshold) for the injured foot at both time points in contrast to sham (Fig. 6a). The brain functional network of SNI animals at day 5 showed no rank order disruption relative to the corresponding day 5 sham group. However, at day 28, SNI animals exhibited group and individual animal reorganization of rank order with a mean *k*_*D*_ value of about −0.3 (Fig. 6b,c). This is a magnitude of disruption similar to the SBP patients at one year, and to the chronic pain patient groups (CBP, OA, and CRPS humans). Consistent with the human *k*_*D*_ values, when we contrasted degree maps between SNI and sham at day 5, no significant differences were observed, while the same contrast at day 28 showed regional increases and decreases in functional connectivity (Fig. 6d). Moreover, in SNI animals at day 28 (but not at day 5), tactile allodynia was correlated with *k*_*D*_ (Fig. 6e), showing that the extent of pain-like behavior in the awake rat is proportional to its degree rank order disruption under anesthesia.

### Potential confounds

One major concern in the study is that head motion artifacts might contribute to differences in connectivity observed between healthy and patient groups. First, we examined and compared head motion displacement in all groups and its relation to *k*_*D*._We found that motion was not significantly different between all patient and healthy groups, and was not correlated to *k*_*D*_ (Supplementary Table 12). In order to investigate the effect of head motion on *k*_*D*_ in a more rigorous manner, we reanalyzed all data presented in Fig. 1 after performing a within-subject, censoring-based artifact removal strategy based on volume censoring, which has been shown to reduce group differences due to motion to chance levels^32^. While volume censoring (scrubbing), substantially decreased regional bias (assessed by examining the correlation of motion with connectivity as a function of distance^33^) between motion and connectivity in all subjects, it did not significantly affect the results (Supplementary Fig. 11). Finally, since chronic pain and depression have a high comorbidity, we investigated the relationship between depression and *k*_*D*._. Overall, depression showed no significant correlation with *k*_*D*_ for all three patient groups examined (Supplementary Table 12).

## Discussion

It is known that a high level of functional interaction between different brain regions is required to support daily cognitive and perceptual activities. In this study, we used noninvasive neuroimaging to construct brain graphs that provide powerful models of the brain connectome^25^,^34^. We measured brain functional connectivity and network properties in various cohorts of patients (at various scales and densities) who are either suffering or transitioning into chronic pain, as well as in an animal model. A key theoretical and clinical question of interest was the nature of any topological abnormality in the brain network organization that might relate to the chronic pain state, which could elucidate which aspects of normal brain network organization might be critical for the development and/or maintenance of chronic pain.

The most important finding was that all clinical cohorts (CBP, SBP, OA and CRPS), as well as rats with SNI injury for 28 days, exhibited a similar disruption of degree rank order, *k*_*d*_, in their functional networks. All groups exhibited a disruption of equivalent magnitude, about 20%, compared to the normative state. In addition, in all individuals (patients or rodents), variations in pain measures were proportional to the extent of degree rank disruption, with higher pain indices always corresponding to a larger *k*_*D*_. The notion of degree rank disruption as a biomarker for the chronic pain state was substantiated by further observations from the human and animal studies. For example, changes in individual *k*_*D*_ were proportional to changes in reported pain intensity over a time period of six months in a subgroup of CBP patients. In addition, the relationship between *k*_*D*_ and pain intensity emerged when pain became chronic and was not manifested in acute or subacute states in both humans and animals. Moreover, we found that *k*_*D*_ accurately predicted pain intensity in two novel groups of CBP and OA patients. Altogether, these results provide compelling evidence that *k*_*D*_ reflects network abnormalities necessary for the manifestation of the chronic pain state in both humans and animals. Remarkably, we could capture the extent of rank order disruption by sampling randomly from as little as 1% of the nodes of the brain network, demonstrating that reorganization of rank ordering permeates the entirety of the brain network in chronic pain.

High-quality evidence points to modularity as one of the most consistent non-random attributes characterizing large-scale, especially biological, networks^26^. Our results show that, while the brain networks can be equally well decomposed into a set of modules in patients and healthy volunteers, the nodes comprising specific modules were markedly variable in the pain patients in proportion to *k*_*D*_ and the magnitude of reported pain. Overall, patients showed lower community structure similarities (about 20%) to healthy controls and an equivalent community structure similarity across types of chronic pain, implying that the organization of community structures in patients diverges from healthy subjects in an undifferentiated pattern. The brain regions most consistently showing community membership divergence also did not differentiate across types of chronic pain. They generally indicated a shift of information away from the somatosensory module to frontal DMN, and from posterior DMN to the attention network, suggesting a shift in the balance between attending to internal and external states. The most consistent earlier results regarding perturbations of resting state point to changes in functional connectivity between the insula and the medial prefrontal cortex and between the insula and the DMN^6^,^12^,^35^. These earlier results seem to be a subset of the main regions we identify as changing module allegiance. Despite the NMI and *k*_*D*_ measurements showing complementary results, we emphasize that the specific modular allegiance reorganization (that minimally impacts on NMI) cannot be directly related to the observed *k*_*D*_ changes, since the latter was conserved after dramatic pruning and with random selection of small subsets of the networks in chronic pain.

While the *k*_*d*_ and modularity analysis identified network reorganization commonly across types of chronic pain, the regional nodal degree analysis identified both chronic pain condition-specific and condition non-specific regional increases and decreases in functional connectivity. For all three chronic pain conditions, nodal degree increases were seen in TH and HIP, while decreases were localized to SMA and LPS. The thalamic region approximates lateral spinothalamic terminations and/or the lateral geniculate (given the spatial resolution, we cannot distinguish between these regions), suggesting enhanced sensory inputs to the cortex, either somatosensory (perhaps nociceptive) and/or visual. The increased connectivity of HIP was localized to the posterior hippocampus and is consistent with recent evidence of its role in pain chronification^36^,^37^. The decreased nodal degree seen in SMA suggests deficits in motor planning, while the decreased connectivity of SPL again implies attentional deficits. The connectivity specifically observed for each chronic pain condition implies condition-unique behavioral and cognitive adaptations, as well as suffering and coping strategies dictating these regional reorganizations.

In close similarity to the results we see in the humans, the SNI animals showed rank order disruption in proportion to tactile allodynia only weeks after the injury. This validates the animal model as relevant to human chronic pain, and establishes a time window for pain chronification in SNI. The time at which we observe rank order disruption, 28 days after SNI, is also the time at which functional connectivity of the striatum reorganizes to the rest of the brain, and dopamine receptor gene expression is decreased within NAc in a larger group of these SNI rats^38^. Remarkably, although the rodent network was studied under anesthesia, we observe rank order disruption of a magnitude closely corresponding to the human chronic pain subjects and in proportion to tactile allodynia. As the pain-like behavior is absent under anesthesia, and tactile allodynia was present at days 5 and 28 after SNI but was only related to *k*_*d*_ at day 28, connectivity disruption cannot be a direct consequence of the behavior. The latter is consistent and complimentary to the results we observe in SBP patients. Therefore, we conclude that rank order disruption reflects the brain network burden of the chronic pain state.

Both in human patients and rodents, we observe that rank order disruption is an emergent phenomenon. In the patients, it seems to require about one year of persistent pain, while in the rat it emerges within one month following a neuropathic injury. Disruption of local nodal connectivity also shows time-dependent emergence, closely paralleling the *k*_*D*_ process in both humans transitioning from SBP to CBP and in the rodents. Thus, processes associated with persistence of pain must be determining – or constraining – factors in this time-dependent transformation of the healthy brain to the chronic state. The localized anatomical and functional reorganizations now described in the transition to chronicity in humans and in rodents^7^,^38^ must propagate to more expansive parts of the brain and eventually percolate throughout the network to create a whole-brain rank order disruption. A corollary conclusion from the temporal dependence of the network reorganization is that acute pain (as we show above) and even persistent pain states of short duration are not sufficient to create the chronic brain functional network state. Therefore, the modularity analysis complements the results we see with rank order disruption and points to a decrease in overall regularity and increased randomness in large-scale topology of the brain network in chronic pain.

A key issue for understanding chronic pain in the context of network reorganization is whether the observed disruptions are localized to a unitary set of brain regions, a chronic pain type-specific functional community structure, or a more homogeneously perturbed network impacting most members. From a classical perspective, pain perception is thought to emerge from the activation/deactivation of a set of unitary and fixed brain regions that receive input from peripheral nociceptors. Consistent with this theoretical framework, there is evidence supporting a spatially unique neurological signature encoding acute pain intensity^39^. A more extreme position purports that the posterior insula is the tissue dedicated to pain perception^40^. On the other hand, brain regions underlying chronic pain intensity seem to vary depending on type, experimental setting (e.g. allodynia or spontaneous pain), and chronicity^41^. In this study, the three methods of analysis (rank order, modularity, and regional connectivity) point to the fact that that the brain network in chronic pain is in an abnormal state, existing in the dual state of being globally perturbed and locally disorganized, a reorganization commonly observed across types of chronic pain as well as in rodents. Moreover, it shows local condition-specific disturbances in functional connectivity. We do not know the direction of the influence, or the specific mechanisms regarding the interaction between local and global disturbances. Yet, the multiple regression analysis shows that *k*_*D*_ is the best predictor of individual subject intensity of pain, only when pain is chronic. Moreover, both *k*_*D*_ and NMI show that a higher intensity of chronic pain is always associated with further disruption of functional connectivity of the entire brain. Future studies are necessary to disentangle between *k*_*D*_ reflecting perception of pain and the cost of the pain perception on cognitive processing. On the other hand, our results strongly suggest that higher intensity chronic pain is always associated with increased randomization of functional connectivity, which must translate to more deficient cognitive processing abilities. Within this viewpoint, the specific regional changes would be reflective of behavioral and cognitive abnormalities uniquely relevant to each type of chronic pain. In close similarity to the results we see in the humans, the SNI animals showed rank order disruption in proportion to tactile allodynia, only weeks after injury.

Regarding the methodology used in the current study, it is important to note that basing our assessment of rank disruption on a control population derived from an off-site data set renders our results robust, resilient to scanner biases and more likely generalizable to chronic pain cohorts at large. Moreover, it provides the opportunity to directly compare these results to other chronic disorders or neurological brain states. The latter is especially important as network analyses are beginning to reveal whole-brain disturbances in many neurological conditions^34^,^42^, and standard tools are necessary to enable direct comparisons of differences in reorganization details across such conditions.

In conclusion, we show that chronic pain is a unique neurological state characterized by both the disruption of whole-brain functional connectivity globally, and the disruption of local connectivity, some of which is unique to the types of chronic pain studied. Therefore, the brain in chronic pain is a far more complex state than a simple persistence of nociceptive barrage. Given that chronic pain is associated with peripheral and central reorganization at the cellular and molecular levels, as well as at the larger-scale regional brain anatomical and functional levels, our findings show that the whole-brain network topology can summarize the impact of all of these changes on the organism, independently from the local connectivity disturbances seen in each type of chronic pain. Overall, these results show that chronic pain encompasses a disconnected “network disease” state of the brain; that its presence and intensity can be determined by the unitary measure of extent of rank order disruption; and, that this measure has the potential of becoming an objective diagnostic marker for chronic pain.

## Online Methods

### Human subjects

Human subjects who participated in this study included 75 healthy subjects (44 females, 31 males; age: mean = 44.16, range = 21–78, s.e.m. = 1.28 years), 40 CBP patients (15 females, 25 males; average age: mean = 48.87, range = 21–62, s.e.m. = 1.29 years), 12 SBP patients (6 females, 6 males; age: mean = 47.00, range = 31–57, s.e.m. = 2.41 years), 40 OA patients (20 females, 20 males; age: mean = 55.37, range = 42–66, s.e.m. = 1.01 years), and 22 CRPS patients (18 females, 4 males; age: mean = 42.41, range = 25–61, s.e.m. = 2.57 years). A subset of the data, including 36 healthy subjects, 18 CBP patients, 19 CRPS patients and 14 OA patients were from previous studies (Baliki *et al*.; Baliki *et al*.). CBP and OA patients were subdivided into two groups each. The first set of patients (25 CBP and 20 OA patients), along with the CRPS and SBP patients, was used in the primary analysis of the paper, while the second group of patients (15 CBP and 20 OA patients) was only used to validate main findings. CBP and OA patients were assigned to the discovery or validation groups at random. We did not divide the CRPS group into a discovery and validation group due to the relatively modest number of patients. All experimental protocols were approved by Northwestern University’s Institutional Review Board committee. All subjects were given informed consent forms and all methods used in the study were carried out in accordance with Northwestern University Institutional Review Board committee guidelines.

All subjects were scanned once, with the exception of the SBP patients and matched healthy volunteers, who were followed across 3 different visits (10 weeks, 6 months, and 12 months after symptom onset), and a subgroup of CBP patients and healthy volunteers who were examined twice (with 6 months between visits). In addition, 129 off-site healthy control subjects (72 females, 57 males; average age: mean = 45.95, range = 20–71, s.e.m. = 1.14 years) were taken from Connectome1000 (Biswal *et al*.^24^). The demographic, pain-related, and scanning parameters for CBP, CRPS and OA patients that were used in the primary analyses are presented in Supplementary Tables 1–3, respectively. The demographic and scanning parameters for the off-site healthy controls are presented in Supplementary Table 4. The demographic and pain-related data for SBP patients are listed in Supplementary Table 9. Lastly, the demographic and pain-related data for the CBP and OA patients used in the validation analysis are listed in Supplementary Tables 11 and 12, respectively.

### Pain and depression parameters

CBP, SBP, CRPS and OA patients completed the Short-Form of the McGill Pain Questionnaire (SF-MPQ)^43^, which includes a visual analog scale (VAS) (0 = no pain, 10 = maximum imaginable pain) and pain duration. The OA patients also filled out the Western Ontario and McMaster Universities Index (WOMAC), which includes subscales for function and stiffness as well as pain^44^. Depression scores for all subjects that participated in the study were assessed using the Beck Depression Inventory (BDI)^45^. All questionnaires were given 1 hour prior to brain scanning. Pain intensity for CBP and CRPS patient populations was assessed from the SF-MPQ. Pain intensity for the OA groups was assessed from the WOMAC, since it has been shown to be more specific and accurate in assessing OA pain^44^.

### Human scanning parameters

For all participants, MPRAGE type T1-anatomical brain images were acquired with a 3T Siemens Trio whole-body scanner with echo-planar imaging (EPI) capability, using the standard radio-frequency head coil with the following parameters: voxel size 1 × 1 × 1 mm; TR = 2500 ms; TE = 3.36 ms; flip angle = 9°; in-plane matrix resolution, = 256 × 256; slices = 160; field of view = 256 mm. Resting state fMRI images were acquired on the same day and the same scanner with the following parameters: Multi-slice T2*-weighted echo-planar images with repetition time TR = 2500 ms, echo time TE = 30 ms, flip angle = 90°, number of slices = 40, slice thickness = 3 mm, in-plane resolution = 64 × 64, number of volumes either 244 or 305. The 40 slices covered the whole brain from the cerebellum to the vertex. Scanning parameters for all patients, healthy subjects and off-site controls used in this study are presented in their respective tables.

### Human fMRI preprocessing and data analysis

The pre-processing of each subject’s time series of fMRI volumes was performed using the FMRIB Expert Analysis Tool (FEAT^46^, www.fmrib.ox.ac.uk/fsl) and encompassed: Discarding the first five volumes to allow for magnetic field stabilization; skull extraction using BET; slice time correction; motion correction; spatial smoothing using a Gaussian kernel of FWHM 5 mm; and high-pass temporal filtering (150 seconds). Several sources of noise, which may contribute to non-neuronal fluctuations, were removed from the data through linear regression. These included the six parameters obtained by rigid body correction of head motion, the global BOLD signal averaged over all voxels of the brain, signal from a ventricular region of interest, and signal from a region centered in the white matter. Overall, all healthy controls and patients exhibited minimal motion artifacts (relative mean displacement <1 mm), and were included in the analysis (Supplementary Table 12).

All preprocessed fMRI data were registered into standard MNI space and multiplied by a common gray matter mask generated from all subjects in the study (this step was performed in order to limit all analyses to a common set of gray matter voxels). It was subsequently down-sampled to yield 5828 regional cortical and subcortical nodes (6 × 6 × 6 mm isometric voxels).

### Brain graph construction

Nodes of the brain functional network were defined using either a (1) whole brain voxel-wise (N = 5,828) or (2) ROI analyses with 2 parcellation schema (480 and 90 ROIs). For the voxel-wise networks, nodes were defined as all voxels within the gray matter. To construct the whole brain voxel-wise connectivity networks for each subject, we first computed the Pearson correlation coefficient (R) for the 16,979,878 possible pairs of the 5,828 cortical and subcortical voxel time series from the preprocessed resting state fMRI data. For each subject, the threshold was calculated to produce a fixed number of edges (M) to be able to compare the extracted graphs^23^. Therefore, the values of the threshold are subject-dependent. Each of these extracted graphs comprised of N = 5,828 nodes corresponding to the number of voxels, and M undirected edges corresponding to the significant nonzero absolute values of correlation greater than the value of the threshold. Since the value of the chosen threshold is important^23^,^47^, we chose to test several values of threshold, from a conservative threshold corresponding to 10% connection density (the percentage of edges with respect to the maximum number of possible edges [(N × N − 1)/2]) to a lenient threshold corresponding to 50% link density. Networks constructed at 10% link density are dubbed sparse networks, while those constructed at 50% are dense. In addition to the subject brain graphs, we also constructed 25 Erdos–Renyi (E–R) random networks with the same number of nodes (N) and edges (M). In contrast to fMRI-based graphs, the edges in the random graphs connecting two nodes are assigned randomly. The random graphs were constructed using the open source brain connectivity toolbox (BCT, available at https://sites.google.com/a/brain-connectivity-toolbox.net/bct/).

ROI-based functional networks were constructed using the same methodology described above. The first ROI parcellation template was derived from cortical and subcortical Harvard-Oxford structural atlases (HOSA; http://www.cma.mgh.harvard.edu/fsl_atlas.html) using previously described methods^10^. Briefly, the HOSA (48 cortical and 21 subcortical regions) were further subdivided into smaller uniform ROIs in proportion to their volumes. To subdivide a HOSA node into K number of ROIs, K voxels were chosen at random within a HOSA ROI. Each of these voxels defined the “origin” of a distinct ROI. The remaining voxels encapsulated by the HOSA ROI were then assigned to one and only one of the K origins as dictated by the shortest Euclidean distance. This guaranteed contiguity of each micro node. The procedure was repeated independently for each HOSA ROI, resulting in 480 cortical and subcortical ROIs. The second ROI parcellation template used was based on the automatic anatomical labeling (AAL) atlas^48^, and constituted all 90 cortical and subcortical regions (cerebellar regions were not included).

### Graph Metrics calculations

Following Bullmore and Sporns (2012), several topological properties of the graphs were computed using the BCT. These include clustering (a measure of information segregation), global efficiency (a measure of information integration), and modularity (a global measure of the near-decomposability of the network into a community structure of sparsely interconnected modules). Each of these measures – except for modularity – is defined at the nodal level. The global average of these metrics was estimated over all nodes in each network, resulting in one measurement per subject. For each subject, we also computed the ‘small-worldness’ based on the tradeoff between clustering and efficiency (Humphries and Gurney, 2008). The network G is said to be a small-world if efficiency_*G*_ ≤ efficiency_*random*_ and clustering_*G*_ ⨠ clustering_*random*_. Thus, small-worldness for any given network G was computed as [(clustering_*G*_/clustering_*random*_)/(efficiency_*random*_/efficiency_*G*_)], where a network is deemed a ‘small-world’ if ratio >2. Graph metrics were computed for all brain networks at various link densities. Differences in topological properties between groups were computed using a repeated measure ANCOVA, with age and gender as covariates of no interest. Pair-wise differences between patient groups and healthy subjects were determined using a Tukey post-hoc test.

### Estimation of the degree rank order disruption Index (*k*_*D*_)

Degree rank order disruption (*k*_*D*_) of individual subjects was evaluated in relation to the normative network topology of the off-site control group *k*_*D*_ following methods described by Achard *et al*.^23^. To construct the degree rank order disruption index, we first subtract the off-site control group mean nodal degree from the degree of the corresponding node in a given individual subject, and then plot this individual difference against the off-site control group mean. This results in a scatter plot of N points, where N = number of nodes in the network. *k*_*D*_ is then defined as the gradient of a straight line fitted to a scatter plot following the linear regression (y = *k*_*D*_ *x + b), where y = nodal degrees of subject – nodal degree of off-site control, x = nodal degree of off-site controls, and b = residual or intercept of the regression. Significant *k*_*D*_ differences between patients and healthy subjects at each link density were computed using a repeated measure ANCOVA with age and gender as covariates of no interest. Pair-wise differences between patient groups and healthy subjects at any given link density were determined using a Tukey post-hoc test. Relationships between *k*_*D*_ and clinical pain parameters in patients were estimated using linear regression. Similar analysis was performed longitudinally for a matched subgroup of CBP and healthy subjects, and between SBP (n = 12) and healthy (n = 12), matched for age and gender, selected from our healthy group for visits 1, 2 and 3 at 10% link density. Longitudinal changes in *k*_*D*_ between patients and healthy subjects were determined using a repeated measure ANCOVA with age and gender as covariates of no interest. Pair-wise differences between patient groups and healthy subjects at any given time were determined using a Tukey post-hoc test.

### Modularity analysis

The measurement of modularity can be determined using various algorithms and approaches, each with its own strengths and weaknesses^49^. Here we used a similar approach as the one described by Cole *et al*.^50^. We used a fast and accurate multi–iterative generalization of the Louvain algorithm^51^, provided and recommended by the BCT. First, we used this function over two parameters (link density and structural resolution) to find the optimal community partition for the off-site control data mean resting-state functional connectivity matrix. The search was conducted across combinations of these two parameters (link densities of 10 to 50% in increments of 10%, and resolution of 1 to 2 in increments of 0.25). In a similar fashion to Cole *et al*.^50^, our parameter selection was based on two criteria: (1) there should be a peak of partition similarity^52^ among adjacent locations in this two-dimensional parameter space, and (2) there should be distinct communities corresponding to visual, default-mode, salience, sensorimotor and attention systems (given the numerous reports of their existence and their relative importance in pain research). The nearest-neighbor similarity peak in parameter space resulted in a six-community at link density = 10%, and spatial resolution = 1.5, which included all modules of interest. Since correlation networks can exhibit modularity degeneracy (the existence of multiple distinct community structures of the same network), we utilized a consensus analysis over 100 repetitions. First, for each network we performed 100 modular partitions at link density = 10%, and spatial resolution = 1.5. This resulted in 100 community structures of the same network. The final community structure of any given network was created by thresholding the averaged within-module connectivity likelihood matrix (generated from the 100 repetitions) at 0.9, meaning that if the likelihood for two nodes belonging to the same module above 0.9 across all 100 repetitions, they were considered in the same module. The final community structure for the off-site control mean functional connectivity matrix included 6 modules (Fig. 3A). The same methods were applied to individual patient and healthy subjects, yielding similar community partitions.

The global similarity between two modular partitions or community structures was quantified using normalized mutual information (NMI) provided by BCT. The NMI ranges from 0 to 1, where 0 signifies that the partitions are totally independent and 1 that they are identical. This pairwise similarity measure is used to assess differences between community detection algorithms^49^. Similarity of community structures between healthy subjects and patients were assessed using NMI in relation to the group average of off-site healthy controls to ensure that outcomes were unbiased. Statistical differences between healthy subjects and patients were assessed using an ANCOVA with age and gender as covariates of no interest. Pairwise group differences between the CBP, CRPS and OA groups compared to healthy individuals were determined using a Tukey post–hoc analysis.

Nodal differences between community structures were determined using a module-allegiance with respect to the group community structure of off-site control data, adapted from Bassett^53^. For each subject, nodes that showed the same modular membership to off-site controls were assigned a value of 1, whereas nodes exhibiting a different membership were assigned a value of 0. This resulted in a binary module-allegiance map for each subject. Group average module-allegiance maps were generated by averaging the individual module-allegiance maps within each group, thus generating maps with nodes values ranging between 0 (all subject within this groups show different community membership for that node compared to the offsite-controls) and 1 (all subject within this group show the same community membership for that node compared to the offsite-controls). Nodes that showed significantly nodal module-allegiance differences across all patient groups and healthy subjects were determined using a whole-brain voxel-wise permutation test in FSL^54^ (p < 0.01, FWE corrected using threshold free cluster estimation).

Group specific changes in the community memberships of the nodes showing significant module-allegiance differences were determined using an ANCOVA with age and gender as covariates of no interest. First, we identified two regions of interest (ROI) from the previous analysis. For each subject, the module-allegiance of either ROI (average module-allegiance for all nodes within the ROI) was determined for the six modules identified from the offsite-control group, resulting in 6 values (0–1), representing the extent of module-allegiance of the ROI to the 6 communities. Group differences between ROI module-allegiance to any given community were determined using an ANCOVA with age and gender as covariates of no interest. Since the analysis was performed 6 times (once for each community), differences were considered significant if the group effect from the ANCOVA exhibited p–value < 0.0083 (0.05 divided by 6). Pairwise group differences between the CBP, CRPS and OA groups compared to healthy individuals were determined using a Tukey post–hoc analysis.

### Calculation of spatial nodal degree differences between patients and healthy controls

To localize the nodes (voxels) that exhibited significant changes in the number of connections (degrees) in patients and our healthy control group, we performed a whole-brain voxel-wise analysis. First, for each subject, we computed the number of edges (degrees) for each node, using the BCT toolbox. We then constructed a single brain volume in standard MNI space for each subject, where the value assigned for each voxel corresponds to the degree of that given voxel. Differences in nodal degree across all subjects (patients and our healthy) subjects, at 10% link density, were carried out using Randomise in FSL (Winkler *et al*.^54^). This technique uses permutation-based inference to allow for rigorous comparisons of significance within the framework of the general linear model. Group differences were tested against 5000 random permutations. The generated statistical maps were corrected for multiple comparisons using threshold-free cluster enhancement (TFCE) family-wise error (FWE) correction (p < 0.05).

Pairwise group differences in nodal degree for the 755 nodes that showed significant group effect were determined using a Tukey post–hoc analysis (corrected for multiple comparisons using FDR p < 0.05). Brain regions that exhibited similar increases or decreases in nodal degree in patients compared to healthy subjects, as well as brain regions that showed specific nodal degree to chronic pain type were determined using a conjunction analysis on the appropriate pair-wise contrast maps.

Finally, we investigated the reproducibility of our results at different link densities (10–50%) using a ROI analysis. First, for each subject, we computed the number of edges for each node, using the BCT toolbox across all densities. The number of edges for each ROI was determined as the mean number of edges of all nodes within the ROI (the ROI’s coordinates and cluster sizes are listed in Supplementary Table 6). Differences between groups and densities and their interaction for each ROI were assessed using a repeated measure ANCOVA with age and gender as covariates of no interest.

### Robustness to noise

Here we investigated the resilience of the *k*_*D*_ measure to noise level. White Gaussian noise (WGN) was introduced to the original bold time series signal at various levels ranging from 20% (80% original signal plus 20% noise) to 100% (0% original signal plus 100% noise) with 20% increments. Binarized brain functional graphs (10% link density) were generated for each subject and noise level. Group and individual *k*_*D*_values for the patients and healthy subjects at different noise levels were generated with respect to the off-site control group (i.e. noise % = 0) as described above. Differences in *k*_*D*_ between groups across all noise levels were assessed using a repeated measure ANCOVA, with age and gender as covariates of no interest. Significant differences in *k*_*D*_ between patient groups and the healthy group were determined using a Tukey post–hoc test.

### Animals

A total of 50 adult male Sprague Dawley rats (Harlan, Indianapolis, IN; 200–250 g) were used in these experiments. Animals were housed on soft bedding in groups of three per cage on a 12-h light/dark cycle in a temperature-controlled environment (21 ± 2°C), with food and water available ad libitum. For all animals, handling and testing were performed during the light period. To minimize stress, they were handled regularly before injury and before behavioral testing. All experimental procedures were approved by the Northwestern University Institutional Animal Care and Use Committee, and all experiments were conducted in accordance to the approved guidelines. Behavioral measures and initial fMRI data analyses were performed in a blinded fashion.

Animals were divided into two groups: the short term group, tested 5 days after induction of nerve injury, and the long term group, tested 28 days after injury (day 5: SNI = 12, sham = 12 animals; Day 28: SNI = 13, sham = 13 animals). Because physiological recording was used for fMRI data pre-processing, rats with incomplete physiological recordings during the scans were excluded from the fMRI study. These include one SNI animal at day 5 and 2 sham animals at day 28.

### Spared Nerve Injury (SNI)

SNI was used as an animal model of persistent peripheral neuropathic pain. The SNI model has been described previously^30^. Animals were anesthetized with isoflurane (1.5–2%) and a mixture of 30% N_2_O and 70% O_2_. The sciatic nerve of the left hind leg was exposed at the level of trifurcation into the sural, tibial, and common peroneal nerves. The tibial and common peroneal nerves were tightly ligated and severed, leaving the sural branch intact. Animals in the sham injury group served as the control as their sciatic nerves were exposed, as in the SNI procedure, but they received no further manipulations.

### Assessment of mechanical allodynia

Tactile sensitivity of the hind paw was measured using withdrawal responses to a series of von Frey filaments. Animals were placed in a Plexiglass box with a wire grid floor and allowed to habituate to the environment for 10–15 minutes. Filaments of varying forces (Stoelting Co, USA) were applied to the plantar surface of the hind paw. Filaments were applied in either ascending or descending strengths to determine the filament strength closest to the hind paw withdrawal threshold. Each filament was applied for a maximum of 2 seconds at each trial; paw withdrawal during the stimulation was considered a positive response. Given the response pattern and the force of the final filament, 50% response threshold (in grams) was calculated^55^. Differences in mechanical threshold between SNI and sham at days 5 and 28 post surgery were compared using an unpaired t-test.

### Animal scanning parameters

All MR experiments were carried out on a Bruker 7 T/40 cm horizontal magnet (Clinscan, Bruker Biospin, Ettlingen, Germany) with a surface coil. Blood oxygen level-dependent (BOLD) contrast-sensitive T2*-weighted gradient-echo echo-planar images were acquired for resting state fMRI scans. Each scan consisted of 300 volumes of 14 slices acquisition (repetition time (TR) of 1.3 seconds, echo time (TE) of 25 milliseconds, flip angle = 60°, 1.0 mm slice thickness, and 0.5 × 0.5 mm in-plane resolution). A high-resolution T2-weighted RARE anatomical reference was acquired for each animal (1.0 mm slice thickness and 0.273 × 0.273 mm^2^ in-plane resolution). An additional T2-weighted RARE anatomical scan with the same geometry as the functional image (1 mm slice thickness and 0.5 × 0.5 mm in-plane resolution) was also acquired and used as a low-resolution anatomical reference.

Anesthesia was induced and maintained during the experiments with isoflurane (1.75–2.5%) mixed with air. Body temperature, respiratory rate, and heart rate were monitored and recorded during scans (Model 1025; SA Instruments, Stony Brook, NY, USA). The monitoring system was operated using a fiber optic temperature probe, respiration pad and fiber optic pulse oxymeter. Respiratory and cardiac waves were recorded during image acquisition with a temporal resolution of 0.001 kHz. To maintain body temperature around 37 °C during the imaging session, a feedback-controlled water circulation system (Medres, Cologne, Germany) was used to heat the base of the cradle. Resting state fMRI scans were collected only when physiological parameters remained stable for about 10 minutes.

### Animal fMRI preprocessing and data analysis

The resting state fMRI data was preprocessed with AFNI (http://afni.nimh.nih.gov) and FSL 5.1 (FMRIB’s Software Library, http://www.fmrib.ox.ac.uk/fsl). The fMRI were first skull stripped and preprocessed using AFNI by applying de-spiking, removal of physiological artifacts from respiration and heartbeat, and correction for slice timing. Rats that had incomplete respiratory and cardiac recordings were discarded from further analysis. The images were then processed using FSL for correction for motion, spatially smoothed with a Gaussian kernel of 0.8 mm FWHM and high pass filtered with a cutoff of 100 seconds.

Volumes from functional images were registered to a standard space with a three-step process. Images were first aligned with the individual’s low-resolution anatomical image followed by alignment with the individual’s high-resolution anatomical image, and then co-registered to a standard space. Similar to the human preprocessing analysis, several sources of noise were removed from the data through linear regression. These included the six parameters obtained by rigid body correction of head motion, the global BOLD signal averaged over all voxels of the brain, signal from a ventricular region of interest, and signal from a region centered in the white matter. Following preprocessing and registration into standard space, resting state fMRI scans were down sampled to yield 11567 isometric (0.4 × 0.4 × 0.4 mm) voxels.

### Estimation of the degree rank order disruption Index (*k*_*D*_) in animals

Degree rank order disruption, *k*_*D*_, of individual or group animals (SNI or sham) was evaluated in relation to the mean degree map of the time-matched sham group (that is for day 5 SNI or sham, *k*_*D*_ was evaluated relative to a mean degree map of all day 5 sham animals, n = 12; similarly, for day 28 animals, *k*_*D*_ was measured relative to day 28 sham group mean degree map, n = 11), following methods described by Achard *et al*.^23^. This procedure was adopted to minimize differences due to physiological (body weight and age) factors, as well as scanner-related artifacts such as level of anesthesia and variability in the magnetic field between the two different sets of experiments performed on days 5 and 28. Significant *k*_*D*_ differences between sham and SNI animals at 10% link density were computed using a two-sided t-test between sham and SNI animals at days 5 and 28 independently. The relationship between *k*_*D*_ and mechanical thresholds in SNI animals was estimated using linear regression analysis.

### Calculation of spatial nodal degree differences between SNI and sham animals

Differences in nodal degree between sham and SNI animal were determined using a whole-brain voxel-wise analysis similar to that of human subjects. First, for each animal, we computed the number of edges for each node, using the BCT toolbox. The number of degrees was used to construct single brain volume in standard rodent space for each animal, where the value assigned for each voxel corresponds to its nodal degree. Differences in nodal degree between sham and SNI animals, at 10% link density, were carried out using Randomise in FSL^54^. The resultant statistical maps were FWE corrected using threshold-free cluster enhancement method.

## Additional Information

**How to cite this article**: Mansour, A. *et al*. Global disruption of degree rank order: a hallmark of chronic pain. *Sci. Rep*. **6**, 34853; doi: 10.1038/srep34853 (2016).

## Supplementary Material

Supplementary Information
