# Supplementary material for: Global disruption of degree rank order: a hallmark of chronic pain
Source: Sci Rep. 2016 Oct 11;6:34853. doi: 10.1038/srep34853 (PMC5057075; doi:10.1038/srep34853)
Supplement: Supplementary Information [file srep34853-s1.pdf]

Supporting information for

Global disruption of degree rank order: a hallmark of chronic pain

Authors: Ali Mansour[1], Alex T. Baria[1], Pascal Tetreault[1], Etienne Vachon-Presseau[1], Pei-Ching Chang[1], Lejian Huang[1], A. Vania Apkarian[1]* and Marwan N. Baliki[2,3]*

Affiliations:
[1]Department of Physiology, Northwestern University, Feinberg School of Medicine, Chicago, Illinois 60611, USA.
[2]Department of Physical Medicine and Rehabilitation, Northwestern University, Chicago, Illinois 60611, USA.
[3]Rehabilitation Institute of Chicago, Chicago, Illinois 60611, USA.

*Correspondence to A. Vania Apkarian, email: a-apkarian@northwestern.edu and to Marwan N. Baliki, email: marwanbaliki2008 @u.northwestern.edu

Supplementary information includes:

Supplementary Figs. 1 - 11
Supplementary Tables 1 - 13
Supplementary References

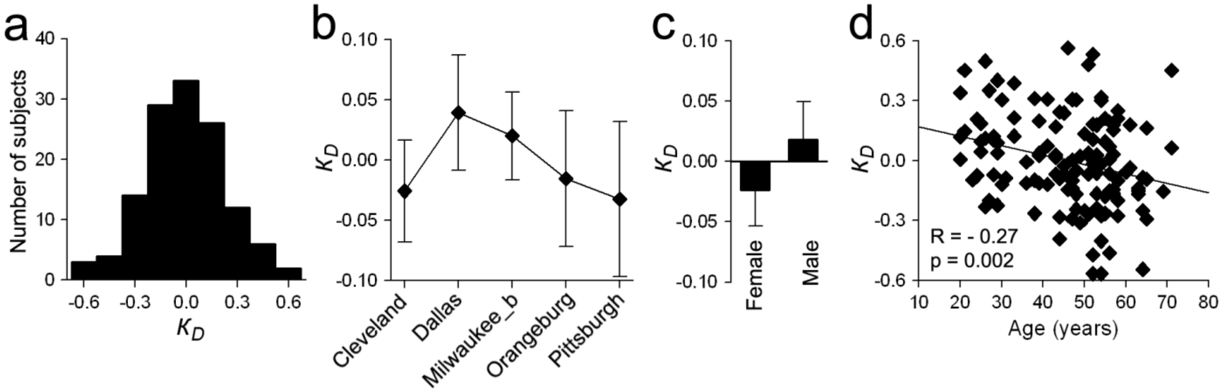

Supplementary Fig. 1. Variability of degree rank order disruption (k_D) in relation to site, age and gender for the off-site connectome1000 control group. Graph shows the distribution of k_D for all off-site control subjects (n =129) used in the study, relative to the group mean degree map. k_D showed a normal (Gaussian) distribution around 0. Relationship of k_D with data source site, gender and age was investigated using a ANCOVA. **(b)** Plot shows the mean ± s.e.m for k_D for each site, relative to the group mean degree map. k_D did not exhibit any significant site effect ($F_{(4,122)} = 0.42$, p = 0.80). **(c)** Bar graph represents the mean ± s.e.m for k_D for females and males. k_D did not exhibit any significant gender effect ($F_{(1,122)} = 1.00$, p = 0.30). **(d)** Scatter plot shows the relationship between age and k_D across all subjects. k_D showed a significant relationship with age (R = -0.27, p = 0.002).

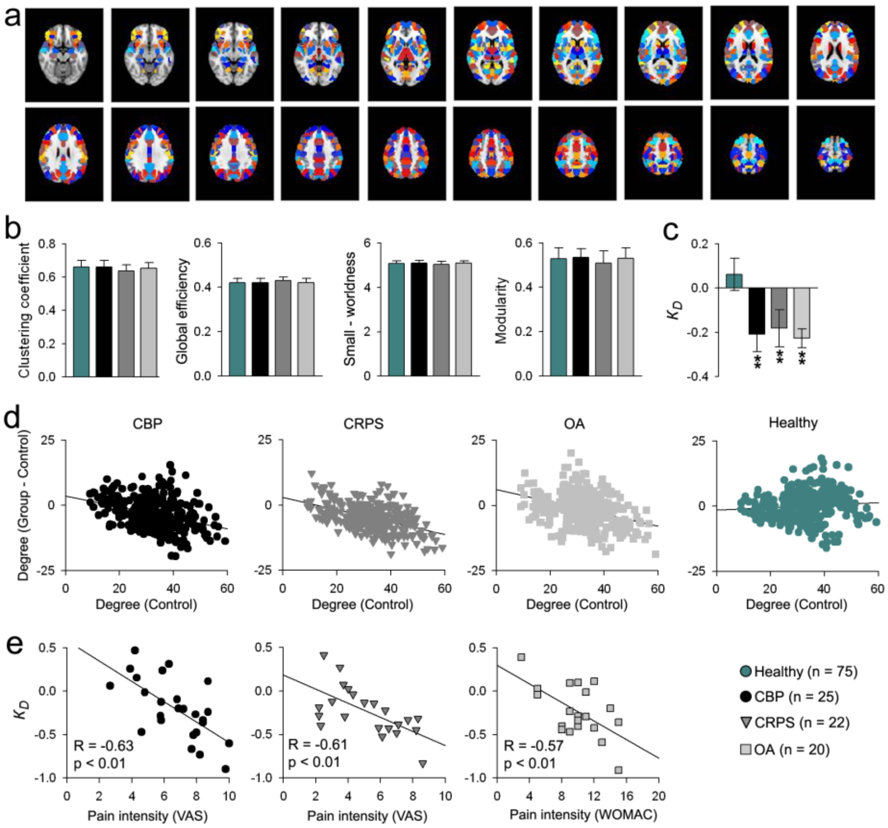

Supplementary Fig. 2. Effect of parcellation scale on degree rank order disruption in chronic pain. (a) Brain networks were constructed from a 480 ROI template derived from a cortical and subcortical Harvard-Oxford structural atlas for 10 % link density. **(b)** Bar graphs show mean ± s.e.m of global graph properties for all groups**.** Global graph properties were not different between healthy subjects and patient groups regarding: clustering coefficient ($F_{(3,136)}$ = 1.74, p = 0.16), global efficiency ($F_{(3,136)}$ = 1.57, p = 0.20), small–worldness ($F_{(3,136)}$ = 1.56, p = 0.20), and modularity ($F_{(3,136)}$ = 1.84, p = 0.12). Differences between groups were computed using ANCOVA analysis with age and gender as covariates of no interest. **(c)** Bar graph shows the mean ± s.e.m of the degree rank order disruption (k_D) computed relative to the off-site healthy control group. Patients exhibited significant degree rank order disruption compared to our healthy controls (group effect: $F_{(3,136)}$ = 6.29, p = 0.0005, ANCOVA with age and gender as covariates of no interest), with CBP (-0.21 ± 0.07), CRPS (-0.18 ± 0.08) and OA (-0.22 ± 0.04) showing significantly lower k_D compared to our healthy control group (0 .06 ± 0.04; *p < 0.05; **p < 0.01, Tukey post - hoc). **(d)** Scatter plots depict the group k_D for CBP, CRPS, OA and our healthy control compared to the off-site control group, for 10% link density. **(e)** Scatter plots show the relationship between k_D and pain intensity at the time of the scan for all patients groups. k_D showed a significant relationship to pain intensity in CBP (R = -0.63, p < 0.001), CRPS (R = -0.61, p = 0.006) and OA (R = -0.57, p = 0.03) for the 10% link density analysis. Finally, we also computed k_D using the 90 ROI AAL template for 10 % link density (data not shown). Similarly to the results listed above, patients exhibited significant degree rank order disruption compared to our healthy controls (group effect: $F_{(3,136)}$ = 5.33, p = 0.003, ANCOVA with age and gender as covariates of no interest), with CBP (-0.19 ± 0.06), CRPS (-0.17 ± 0.09) and OA (-0.20 ± 0.06) showing significantly lower k_D compared to our healthy control group (0.05 ± 0.07). Furthermore, k_D and pain intensity at the time of the scan for all patient groups showed a significant relationship to pain intensity in CBP (R = -0.59, p < 0.01), CRPS (R = -0.55, p < 0.01) and OA (R = -0.58, p < 0.05).

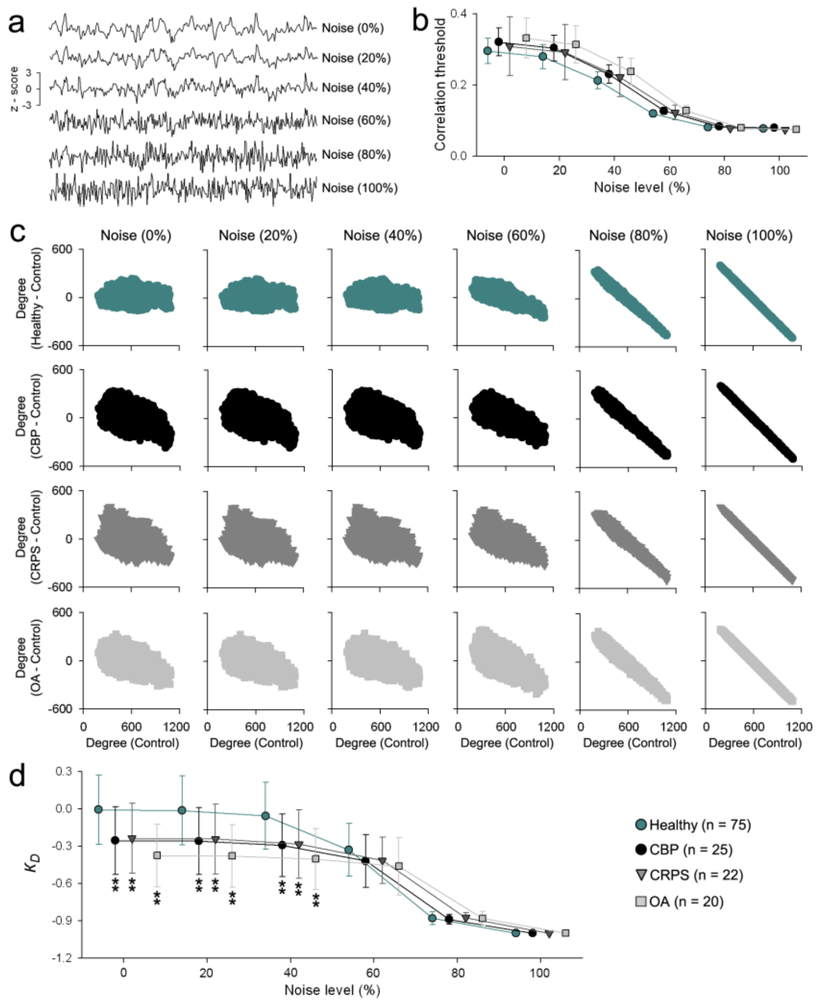

Supplementary Fig. 3. Effect of noise on degree rank order disruption. (a) Example from a single voxel bold signal in which white Gaussian noise (WGN) was introduced to the original bold time series at various levels ranging from 20% (80% original signal plus 20% noise) to 100% (0% original signal plus 100% noise) with 20% increments. Noise level of 0% denotes the original signal (**b)** Graph depicts the mean ± s.e.m. of correlation values required to produce binary functional connectivity maps of 10% link density for CBP, CRPS, OA and our healthy controls, at the various noise levels. For all groups, there was a huge noise effect ($F_{(5,680)} = 189.53$, $p < 0.0001$, repeated measure ANCOVA, with age and gender as covariates of no interest). For noise levels > 60%, threshold had to be set to an r-value < 0.1. **(c)** Scatter plots depict the group k_D for CBP, CRPS, OA and our healthy group compared to the off-site control group for 10% link density at various noise levels. The addition of noise resulted in an artificial k_D only when the noise constituted ≥60% of the original bold signal. **(d)** Mean ± s.e.m of individual k_D values for CBP, CRPS, OA and our healthy subjects. All groups showed stable individual k_D values up to noise level of about 60%, which subsequently decreased with increased WGN (group effect: $F_{(3,136)} = 9.96$, $p = 0.0001$, group by noise interaction: $F_{(15,680)} = 12.10$, $p < 0.0001$, repeated measure ANCOVA). Significant differences in k_D values between patients and healthy subjects at different noise levels were computed using Tukey post-hoc tests (*$p < 0.05$; **$p < 0.01$, compared to our healthy control group). It is important to note that we performed a similar analysis for graphs constructed at 20% link density. We found that an addition of 20% noise was sufficient to distort k_D measurements (data not shown). These results indicate that the resilience of 10% networks to WGN is probably due to high correlation cut-offs and the sparsity of the network.

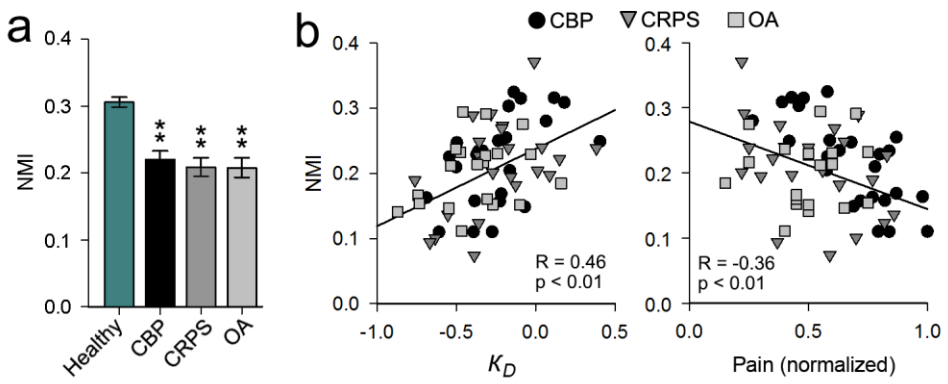

Supplementary Fig. 4. Modular changes in patients reflects globally altered connectivity.
We assessed the contribution of the localized nodal module-allegiance changes within the insular and lateral parietal regions (**Figure 2e**) to the overall modular changes observed in patients. **(a)** Mean ± s.e.m normalized mutual information (NMI) for all groups measured with respect to the mean off-site healthy control after removing the 135 insular and 35 lateral parietal nodes that exhibited significant differences in module-allegiance in patients. NMI still showed a significant group effect ($F_{(3,136)}$ = 25.85, p < 0.0001, ANCOVA with age and gender as covariates of no interest), with CBP (0.22 ± 0.01), CRPS (0.20 ± 0.01) and OA (0.21 ± 0.01) exhibiting lower NMI compared to the healthy group (0.30 ± 0.01) (*p < 0.05; **p < 0.01, Tukey post-hoc compared to healthy). **(b)** NMI of all patients with respect to the mean off-site control showed a significant correlation to k_D (r = 0.46, p < 0.001) and to pain intensity (r = -0.36, p < 0.01). These results indicate that overall changes in community structure observed in patients are mostly driven by nodal memberships throughout the network, and not by localized changes in the insula and parietal regions, which showed similar patterns of module-allegiance changes across types of chronic pain.

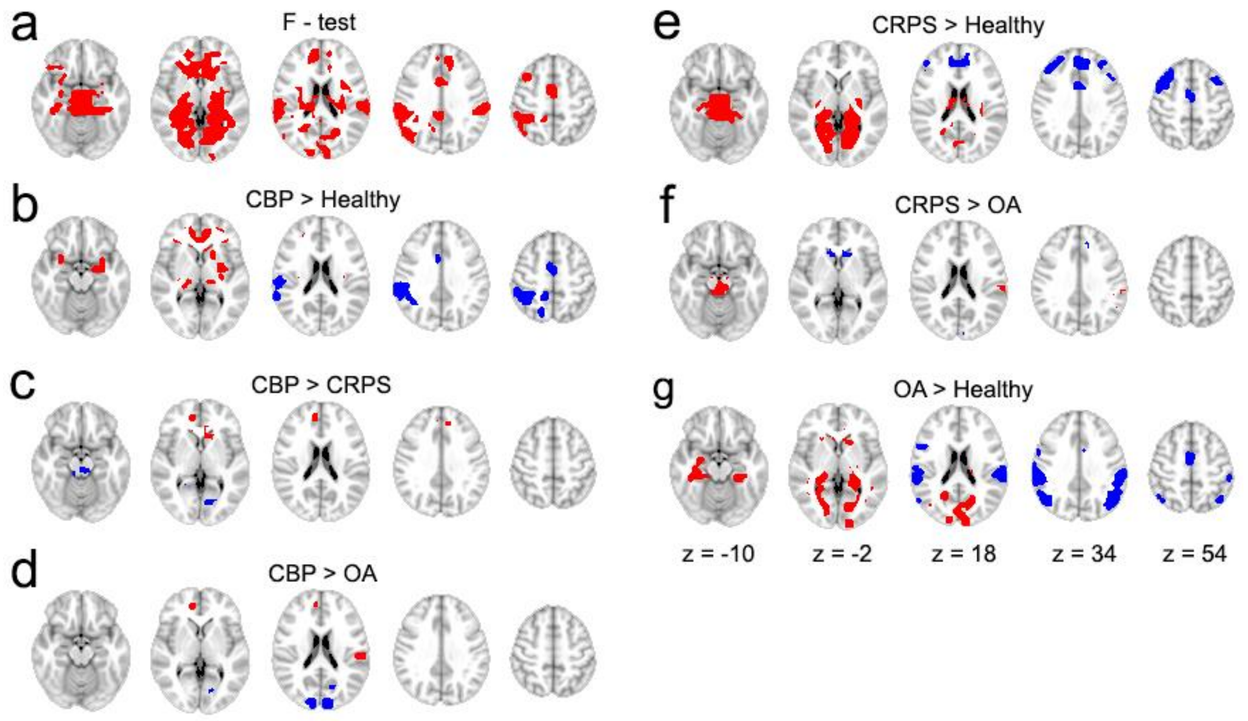

Supplementary Fig. 5. Nodal degree changes in chronic pain patients. (a) Brain slices show regions with significant difference in nodal degree across all groups for 10% link density analysis (whole-brain voxel-wise ANCOVA with age and gender as covariates of no interest, f–zscore > 2.3, p<0.01, FWE corrected using threshold–free cluster enhancement). **(b - g)** Brain slices illustrate the nodes that showed significant differences between all possible pair-wise comparisons (patients and our healthy controls) for 10% link density analysis (Tukey post-hoc, p< 0.05 FDR corrected). Red denotes significantly increased degree and blue denotes significantly decreased degree. Brain regions that showed similar changes in all patients compared to healthy controls (**Figure 3c**) were determined from the conjunction of panels **b**, **e** and **g**. Brain regions that showed CBP-specific nodal degree changes (**Figure 3d**) were determined from the conjunction of panels **b**, **c** and **d**. Brain regions that showed CRPS-specific nodal degree changes (**Figure 3e**) were determined from the conjunction of panels **c**, **e** and **f**. Brain regions that showed OA-specific nodal degree changes (**Figure 3f**) were determined from the conjunction of panels **d**, **f** and **g**. (Coordinates in mm, standard MNI space).

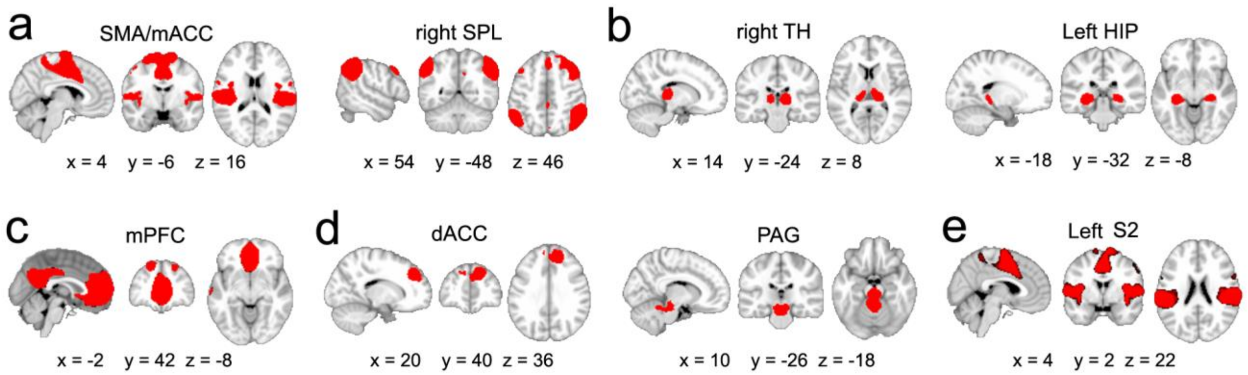

Supplementary Fig. 6. Resting state functional correlation networks for brain regions showing localized nodal degree changes in patients. Brain slices show the average resting-state functional correlation maps (r > 0.2) for the seed regions in a sample of 1000 subjects from Neurosynth [1]. The seed regions were identified from the regions that showed similar and unique nodal degree changes in patients (**Figure 3c-f, Table S6**). **(a)** Brain slices show the functional correlation maps for regions that exhibited common decreases in nodal degree in all patients compared to our healthy subjects. The SMA/mACC region showed extensive connectivity to sensorimotor regions, including bilateral primary and secondary somatosensory regions, motor regions, and posterior portions of the insula. The right SPL showed functional connectivity to brain areas involved in attention, including the posterior parietal regions, parts of the cingulum and right dorsolateral frontal areas. **(b)** Brain slices show the functional correlation maps for regions that exhibited common increases in nodal degree in all patients compared to our healthy subjects. The thalamus connectivity was mainly restricted to the bilateral thalamic nuclei, while the HIP connectivity was mainly localized to the bilateral posterior hippocampus. **(c)** Resting state functional correlation maps for the mPFC seed that exhibited CBP specific nodal degree increases in CBP. The mPFC mainly showed connectivity to brain regions within the DMN. **(d)** The dACC, which exhibited specific nodal degree decreases in CRPS, showed mainly localized connectivity to other frontal regions. On the other hand, the PAG, which showed specific nodal degree increases in CRPS, was mainly connected to brainstem and midbrain regions. **(e)** Resting state functional correlation maps for the left S2 seed that exhibited specific nodal degree decreases in OA. The S2 region showed extensive connectivity to sensorimotor regions, including bilateral primary and secondary somatosensory regions, motor regions and posterior portions of the insula.

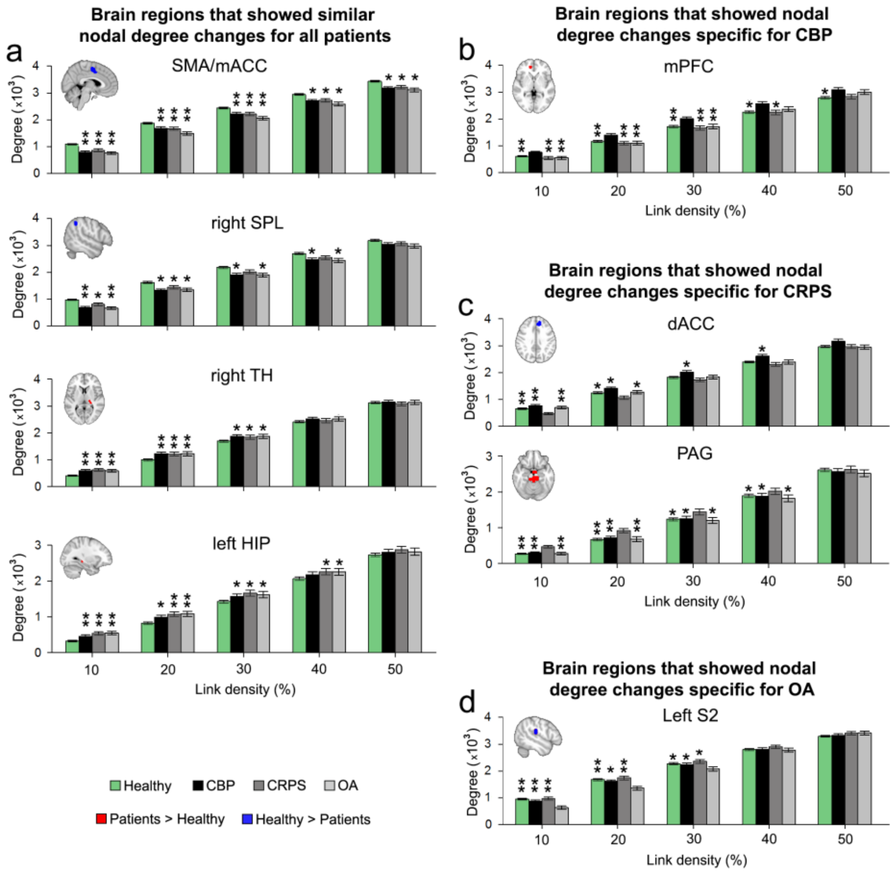

Supplementary Fig. 7. Nodal degree changes in chronic pain patients show consistency across different link densities (a) Bars represent the mean ± s.e.m of nodal degree for the regions that showed similar increases and decreases in patients compared to healthy controls at all link densities examined (10 – 50%). The mACC (group effect: $F_{(3, 136)} = 16.12$, $p < 0.0001$; group x density effect: $F_{(12, 544)} = 1.01$, $p = 0.38$) and right SPL (group effect: $F_{(3, 136)} = 8.26$, $p < 0.0001$; group x density effect: $F_{(12, 544)} = 1.49$, $p = 0.12$) showed decreased nodal degree in all patients compared to healthy subjects. The right TH (group effect: $F_{(3, 136)} = 2.69$, $p = 0.04$; group x density effect: $F_{(12, 544)} = 3.71$, $p < 0.0001$) and left HIP (group effect: $F_{(3, 136)} = 3.29$, $p = 0.02$; group x density effect: $F_{(12, 544)} = 1.90$, $p = 0.03$) showed increased nodal degree in patients compared to healthy subjects mainly for densities ≤< 30% (repeated measure ANCOVA with age and gender as covariates of no interest; *p < 0.05, **p < 0.01, Tukey post - hoc compared to healthy). **(b)** Bars show the mean ± s.e.m of nodal degree for brain regions specifically associated with CBP. The mPFC (group effect: $F_{(3, 136)} = 5.11$, $p = 0.002$; group x density effect: $F_{(12, 544)} = 2.76$, $p = 0.001$) showed increased degree in CBP compared to OA , CRPS and healthy subjects mainly for densities ≤ 30% (repeated measure ANCOVA with age and gender as covariates of no interest; *p < 0.05, **p < 0.01, Tukey post - hoc compared to CBP). **(c)** Bars show the mean ± s.e.m of nodal degree for brain regions specifically associated with CRPS. The dACC (group effect: $F_{(3, 136)} = 3.88$, $p = 0.011$; group x density effect: $F_{(12, 544)} = 3.77$, $p < 0.0001$) showed decreased nodal degree in CRPS compared to CBP, OA and healthy subjects for densities ≤ 20%. On the other hand, the brainstem (group effect: $F_{(3, 136)} = 3.85$, $p = 0.010$; group x density effect: $F_{(12, 544)} = 1.87$, $p = 0.03$) showed increased nodal degree in CRPS compared to CBP, OA and healthy subjects for densities ≤ 40% (repeated measure ANCOVA with age and gender as covariates of no interest; *p < 0.05, **p < 0.01, Tukey post - hoc compared to CRPS) **(d)** Bars show the mean ± s.e.m of nodal degree for brain regions specifically associated with OA. The left S2 (group effect: $F_{(3, 136)} = 2.74$, $p = 0.04$; group x density effect: $F_{(12, 544)} = 8.35$, $p < 0.0001$) showed decreased degree in OA compared to CBP,CRPS and healthy subjects only for densities ≤ 20% (repeated measure ANCOVA with age and gender as covariates of no interest; *p < 0.05, **p < 0.01, Tukey post-hoc compared to OA). (All p – values were adjusted for multiple comparisons using Bonferroni correction)

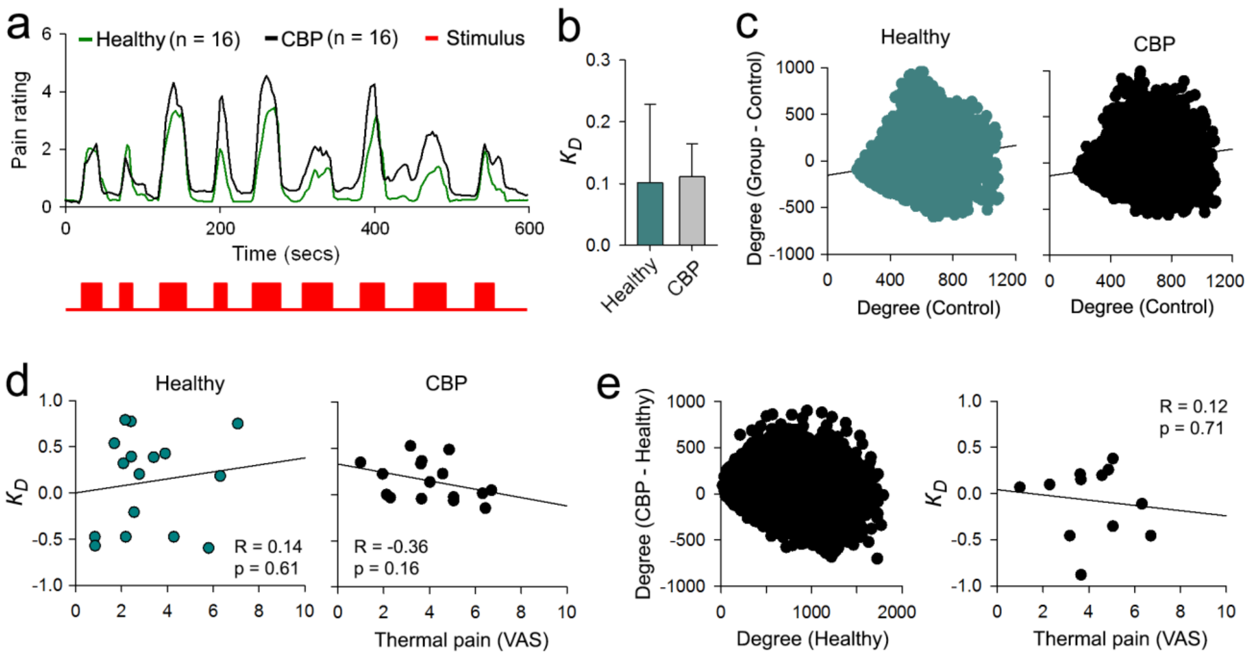

Supplementary Fig. 8. CBP patients and healthy subjects do not show rank order disruption for acute thermal pain. **(a)** Top panel shows average pain ratings for painful heat in CBP (n = 12, black trace) and gender- and age-matched healthy subjects (n =12, green trace) taken from previously published data (see main text). Bottom panel shows the time course of the thermal stimulus (red) applied to the lower back. **(b)** Bar graph shows the mean ± s.e.m k_D for the thermal task in CBP and healthy compared to the off-site control data for 10% link density. There were no differences between the two groups (Healthy: k_D mean ± s.e.m. = 0.10 ± 0.12; CBP: k_D mean ± s.e.m. = 0.11 ± 0.06; $t_{(22)}$ = 0.18, p = 0.84, two-sided unpaired t-test). **(c)** Scatter plots depict the group k_D for healthy subjects (left plot, green circles) and CBP patients (right plot, black circle) compared to the off-site control group for 10% link density. **(d)** Scatter plots show the relationship between k_D and thermal pain intensity at the time of the scan healthy subjects (left plot, green circles) and CBP patients (right plot, black circles). k_D showed no significant relationship with thermal pain intensity in healthy subjects (R = 0.14, p = 0.61) or CBP (R = -0.36, p = 0.16).**(e)** We also computed k_D of functional networks in CBP during thermal task in relation to healthy subjects during the thermal task. CBP did not exhibit any significant k_D compared to healthy subjects during thermal pain (Healthy: k_D mean ± s.e.m. = 0.00 ± 0.09; CBP: k_D mean ± s.e.m. = -0.08 ± 0.11; $t_{(22)}$ = -0.54, p = 0.59, two-sided unpaired t-test). The left scatter plot shows the group k_D for CBP patients during thermal task compared to healthy subjects for 10% link density. The right scatter plot illustrates the relationship between thermal pain intensity and k_D in the CBP group. We did not observe any significant relationship (R = -0.12, p = 0.71).

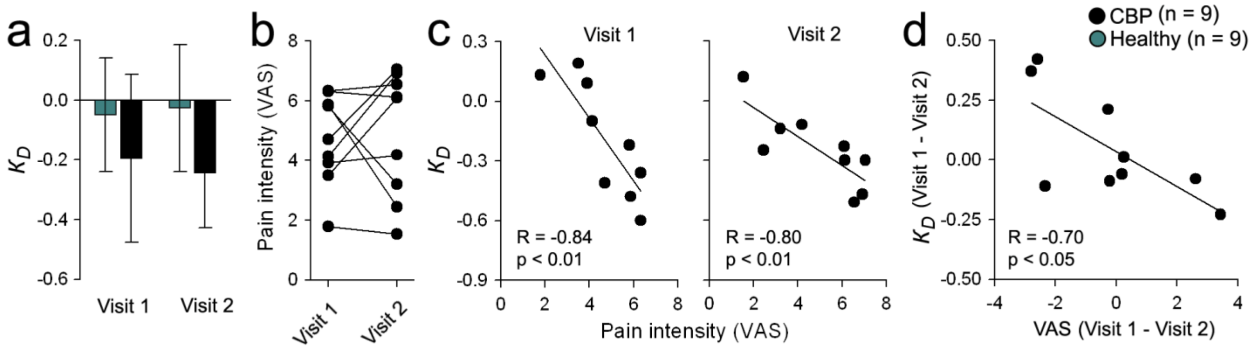

Supplementary Fig. 9. Degree rank order disruption in patients shows temporal reliability over a six-month period (a) Mean ± s.e.m. for k_D in CBP and healthy controls for visit 1 (baseline) and visit 2 (6 months later). The k_D values for all groups were computed in relation to the mean degree map of the offsite healthy controls. On average, CBP showed more negative k_D compared to matched healthy controls ($F_{(1,14)}$ = 3.98, p = 0.061, repeated measure ANCOVA with age and gender as covariates of no interest). **(b)** Graph depicts the individual changes in reported spontaneous pain intensity (gray circles) in CBP patients for visits 1 and 2. Despite the individual variability, the group average pain rating was similar for both visits (paired t - test, $t_{(8)}$ = -0.26, p = 0.79,). **(c)** k_D and back pain intensity exhibited a strong relationship at visit 1 (left scatter, R = -0.84, p =0.005) and visit 2 (right scatter, R = -0.80, p = 0.006). **(d)** Within - subjects changes in k_D showed a significant relationship with corresponding changes in back pain intensity (R = -0.70, p = 0.017).

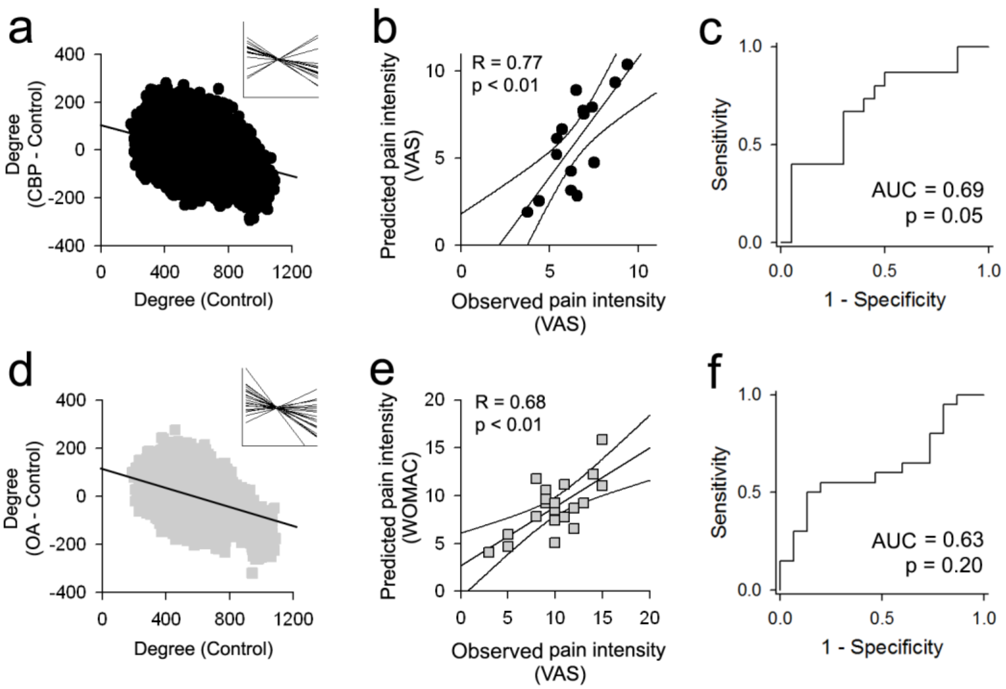

Supplementary Fig. 10. Degree rank order disruption accurately predicts pain intensity in two novel groups of chronic pain patients. Predicted VAS and WOMAC pain intensity scores for the new CBP and OA patients were derived from linear correlations shown in **Fig. 1e**. Accuracy of prediction was assessed using a correlation analysis between the predicted values and the observed values for each group independently. **(a)** Scatter plot shows the degree rank order disruption in CBP (n = 15) for 10% link density. Top right insert shows k_D computed for each CBP patient. Group and individual k_D values were computed with respect to the off-site control group. (**b)** Scatter plot shows association between predicted and observed pain intensity as measured by the VAS scale. Predicted and observed values showed a highly significant relationship (R = 0.77, p = 0.004). **(c)** ROC curve and discrimination probability (AUC, area under the curve) for predicting CBP pain intensity in the novel group of patients based on mPFC nodal degree from **Figure 3d**. Nodal degree of mPFC was able to significantly discriminate CBP from OA patients in the novel group of patients (AUC = 0.69, p = 0.05). **(d)** Rank order disruption in the new OA patients (n =20) for 10% link density. Top right insert shows k_D computed for each OA patient. **(e)** Scatter plot depicts high positive correlation between predicted and observed pain intensity as measured by the WOMAC scale in these OA patients (R = 0.68, p = 0.001). **(f)** ROC curve and discrimination probability for predicting OA pain intensity in the novel group of patients based on S2 nodal degree from **Figure 3f**. Nodal degree of S2 was unable to significantly discriminate OA from CBP patients in the novel group of patients (AUC = 0.63, p = 0.20). ROC curves were also computed using both mPFC and S2 degree in the same model. Nodal degree of mPFC and S2 were able to discriminate CBP and OA patients in the novel group of patients (AUC = 0.72, p =0.012, data not shown).

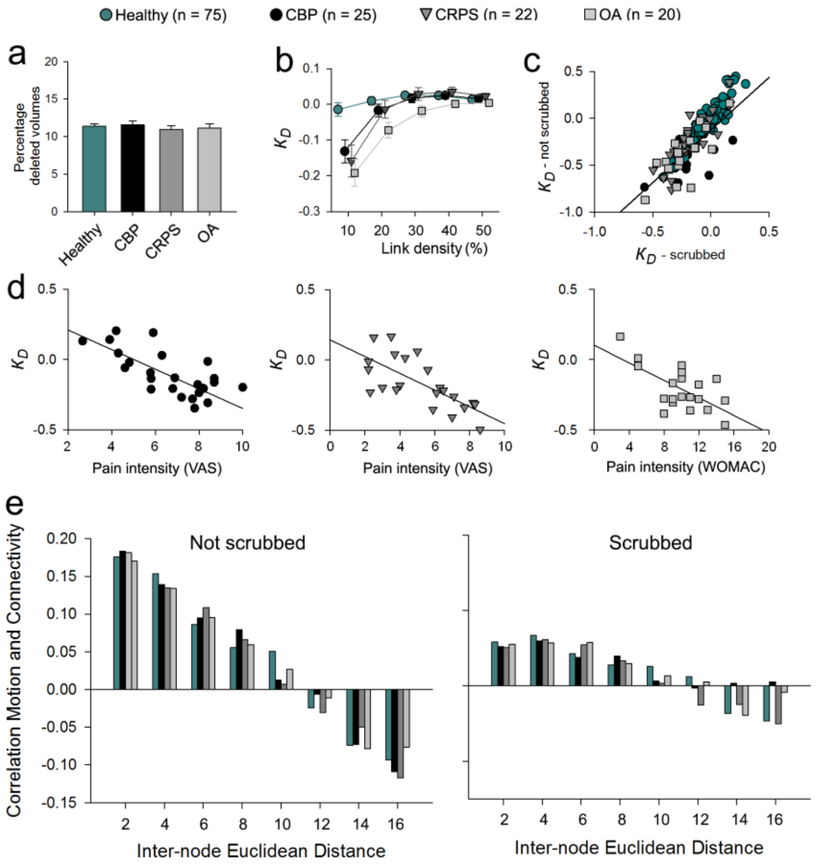

Supplementary Fig. 11. The effect of data scrubbing on degree rank order disruption (k_D). In order to determine the contribution of head motion to k_D, we reanalyzed all data presented in **Fig. 1** after performing a within-subject, censoring-based artifact removal strategy based on volume censoring following the exact procedures described by Power et al [2]. Framewise displacement (calculated as the sum of the absolute values of the differentiated realignment estimates at every timepoint), DVARS (calculated as the root mean square value of the differentiated BOLD timeseries within the whole brain at every timepoint), and the standard deviation of the BOLD signal across all voxels within the brain at every timepoint were used to identify brain volumes with high noise that were subsequently removed during preprocessing for all offsite controls, healthy subjects and patients. Individual subjects' k_D values were then recomputed as described in the main manuscript. **(a)** Bar graphs show mean ± s.e.m of percentage of number of timepoints removed by scrubbing. Overall, around 10% of the time series were removed for healthy and all patient groups ($F_{(3,136)} = 0.29$, $p = 0.83$). **(b)** Mean ± s.e.m. of k_D in our healthy subjects and patients across all link densities. Similar to our original observations, patient groups exhibited significant degree rank order disruption compared to off-site healthy controls (group effect: $F_{(3,136)} = 5.93$, $p < 0.001$), with the most significant differences observed for link density = 10 %. **(c)** k_D values (link density = 10%) before and after scrubbing show high correlation across all subjects ($R = 0.88$, $p < 0.0001$). **(d)** k_D still showed a significant relationship to pain intensity in CBP ($R = -0.74$, $p < 0.001$), CRPS ($R = -0.70$, $p < 0.001$ and OA ($R = -0.64$, $p = 0.002$), at 10% link density. **(e)** Bar graph shows the relationship between motion and connectivity (correlation coefficient) as a function of inter-node distances for our healthy controls and all patient groups. Similar to previous results, motion tends to increase connectivity for adjacent nodes and decrease connectivity between distant nodes (left plot). This relationship was significantly reduced after scrubbing (right plot). Group differences between the motion and connectivity correlation at different distances were examined using the Fisher exact test. There were no significant differences between any pair of groups at any given distance (all p-values > 0.22). The results show that a more aggressive correction for motion and noise still preserves rank order disruption in all patient groups.

Subject	Volumes	TR (seconds)	Slices	Age (years)	Gender	BDI	Pain duration (years)	VAS
CBP01	305	2.5	40	51	F	15	1	8
CBP02	305	2.5	40	62	M	1	38	8.7
CBP03	305	2.5	40	49	F	12	14	8.4
CBP04	305	2.5	40	62	F	13	2	8.4
CBP05	305	2.5	40	60	F	8	30	10
CBP06	305	2.5	40	46	M	13	11	5.9
CBP07	305	2.5	40	49	M	4	41	6.3
CBP08	305	2.5	40	32	M	5	12	7.8
CBP09	305	2.5	40	41	M	3	15	7.7
CBP10	305	2.5	40	48	M	12	1	8.7
CBP11	305	2.5	40	44	M	0	18	9.8
CBP12	244	2.5	40	42	M	2	15	5.8
CBP13	244	2.5	40	39	F	0	10	4.6
CBP14	244	2.5	40	56	F	0	11	4.3
CBP15	244	2.5	40	47	M	0	32	7.94
CBP16	244	2.5	40	58	M	10	20	6.89
CBP17	244	2.5	40	52	M	0	1	4.2
CBP18	244	2.5	40	41	F	0	25	3.9
CBP19	244	2.5	40	49	F	0	7	5.8
CBP20	244	2.5	40	52	M	4	37	7.2
CBP21	244	2.5	40	52	F	5	15	2.7
CBP22	244	2.5	40	48	M	2	10	4.8
CBP23	244	2.5	40	40	F	10	7	6.8
CBP24	244	2.5	40	48	F	19	10	8.21
CBP25	244	2.5	40	21	F	19	11	5.8

Table S1. Demographics, scan parameters and pain characteristics of CBP subjects studied. BDI is Beck's Depression Index, which is higher than healthy subjects, but all CBP would be classified as moderate or low depression. VAS is visual analog scale. It is used to determine intensity of back pain (0-10 scale).

Subject	Volumes	TR (seconds)	Slices	Age (years)	Gender	BDI	Pain duration (years)	VAS
CRPS01	305	2.5	40	61	F	6	0.25	8.2
CRPS02	305	2.5	40	61	M	4	9.75	8.6
CRPS03	305	2.5	40	56	M	12	0.25	2.2
CRPS04	305	2.5	40	48	M	13	1.42	2.5
CRPS05	305	2.5	40	40	M			4.0
CRPS06	305	2.5	40	25	F	13	1.92	6.3
CRPS07	305	2.5	40	26	F	13	0.42	2.2
CRPS08	305	2.5	40	26	F	8	1	4.3
CRPS09	305	2.5	40	27	F			3.5
CRPS10	305	2.5	40	28	F	7	1.6	2.3
CRPS11	305	2.5	40	31	F	5	0.25	3.8
CRPS12	305	2.5	40	32	F	5	1.75	6.1
CRPS13	305	2.5	40	39	F	9	1.33	6.5
CRPS14	305	2.5	40	40	F	10	13.5	8.3
CRPS15	305	2.5	40	41	F			5.9
CRPS16	305	2.5	40	43	F	14	5.08	5.6
CRPS17	305	2.5	40	47	F	13	7.33	7.1
CRPS18	305	2.5	40	48	F			3.0
CRPS19	305	2.5	40	50	F			5.0
CRPS20	305	2.5	40	53	F	5	0.25	7.7
CRPS21	305	2.5	40	59	F			7.0
CRPS22	305	2.5	40	52	F			3.7

Table S2. Demographics, scan parameters and pain characteristics of CRPS subjects studied. BDI = Beck's Depression Index; VAS = visual analog scale

Subject	Volumes	TR (seconds)	Slices	Age (years)	Gender	BDI	Pain duration (years)	VAS	WOMAC (pain)
OA01	305	2.5	40	49	M	9	10	7.1	8.0
OA02	305	2.5	40	53	M	7	36	8.3	5.5
OA03	305	2.5	40	66	M	1		2.1	5.0
OA04	305	2.5	40	51	M	22	14	9.0	7.5
OA05	305	2.5	40	56	F	13	6	5.6	5.0
OA06	305	2.5	40	42	M	9	5	5.7	9.50
OA07	305	2.5	40	53	M	0	11	7.0	12.0
OA08	305	2.5	40	52	F	14	4	8.8	6.5
OA09	300	2.5	40	51	M	14	1	7.0	7.0
OA10	300	2.5	40	57	F	10	6	5.0	11.0
OA11	300	2.5	40	52	F	10	2	8.0	9.0
OA12	300	2.5	40	62	F	6	5	7.0	4.5
OA13	300	2.5	40	48	M	9	10	7.0	12.0
OA14	300	2.5	40	56	F	2	6	5.0	5.0
OA15	300	2.5	40	53	M	2	30	6.0	7.0
OA16	300	2.5	40	64	F	7	12	8.0	7.5
OA17	300	2.5	40	54	F	0	25	9.0	10.0
OA18	300	2.5	40	45	M	1	1.5	7.0	7.0
OA19	300	2.5	40	61	M	3	3	7.0	10.0
OA20	300	2.5	40	58	M	8	6	6,0	8.0

Supplementary Table 3. Demographics, scan parameters and pain characteristics of OA subjects studied. BDI = Beck's Depression Index; VAS = visual analog scale; WOMAC = Western Ontario and McMaster Universities Osteoarthritis Index

Dataset	Number of subjects	Volumes	TR (seconds)	Slices	Age (years)	Gender
Cleveland	30	127	2.8	31	43.23 ±11.18	11 M / 19 F
Dallas	23	115	2.0	36	42.21 ± 20.41	11 M / 12 F
Milwaukee_b	46	175	2.0	64	53.58 ± 5.79	15 M / 31 F
Orangeburg	17	165	2.0	22	40.59 ± 10.99	12 M / 5 F
Pittsburgh	13	275	1.5	29	38.84 ± 9.41	7 M / 6 F

Supplementary Table 4. Demographics, scan parameters for the off-site connectome1000 control group. TR = time to repetition; M = male; F = female. Age reported as mean ± standard deviation**.**

% Link density	Healthy	CBP	CRPS	OA
10	0.00 ± 0.03	-0.25 ± 0.05 **	-0.21 ± 0.06**	-0.36 ± 0.06**
20	0.03 ± 0.04	-0.14 ± 0.06*	-0.08 ± 0.08*	-0.23 ± 0.07**
30	0.10 ± 0.04	0.00 ± 0.07	0.09 ± 0.08	-0.06 ± 0.08
40	0.14 ± 0.05	0.11 ± 0.08	0.17 ± 0.09	0.04 ± 0.09
50	0.13 ± 0.05	0.18 ± 0.08	0.20 ± 0.09	0.09 ± 0.10

Supplementary Table 5. Chronic pain is associated with degree rank order disruption (k_D) of whole brain functional networks. Table lists the mean ± s.e.m of k_D for all the groups at all link densities studied (10 - 50%). Individual k_D values were computed with respect to the mean off-site healthy controls. 'Healthy' are our own controls relative to off-site control. Differences between groups were determined using a repeated measure ANCOVA with age and gender as covariates of no interest. Differences between patients and healthy subjects were determined using a Tukey post – hoc. All patient groups showed significant degree rank order disruption compared to healthy at 10 and 20% link densities (*$p < 0.05$; **$p < 0.01$, Tukey post - hoc compared to healthy).

Region	Condition	Cluster size	Coordinates (mm)
SMA/mACC	Decreased nodal degree (all patients)	14 nodes	x = 4, y = -2, z = 48
Right SPL	Decreased nodal degree (all patients)	3 nodes	x = 54, y = -48, z = 44
Right TH	Increased nodal degree (all patients)	14 nodes	x = 22, y = -28, z = 6
Left HIP	Increased nodal degree (all patients)	5 nodes	x = -28, y = -30, z = -6
mPFC	Increased nodal degree (CBP specific)	3 nodes	x = -6, y = 48, z = -2
dACC	Decreased nodal degree (CRPS specific)	2 nodes	x = 14, y = 42, z = 36
PAG	Increased nodal degree (CRPS specific)	4 nodes	x = 0, y = -28, z = -18
Left S2	Decreased nodal degree (OA specific)	3 nodes	x = -14, y = 16, z = -2

Supplementary Table 6. Anatomical localization of nodal degree changes. Table lists the cluster size (number of nodes) and MNI coordinates (mm) for the brain regions that showed similar and unique functional connectivity changes in CBP, CRPS and OA.

Independent parameter	Correlation	P - value
Age	0.171	0.197
Gender	0.058	0.663
Duration	0.256	0.06
BDI	-0.110	0.407
k_D	-0.503 **/++	<0.0001
NMI	-0.378 **/+	0.003
Clustering coefficient	-0.098	0.459
Global efficiency	0.024	0.856
Degree mACC	-0.30 *	0.022
Degree right SPL	-0.21	0.117
Degree right TH	0.44 **/++	<0.0001
Degree left HIP	0.31*	0.018
Degree mPFC	0.18	0.176
Degree dACC	-0.03	0.847
Degree brainstem	0.27*	0.037
Degree left S2	-0.16	0.235

Supplementary Table 7. Correlation of chronic pain intensity with demographic, clinical, and brain network parameters. Table lists the correlation of pain intensity across all patients (n = 67) with various parameters. All CBP (n = 25), CRPS (n = 22) and OA (n = 20) patients were included in the analysis (*p<0.05, **p<0.01 uncorrected; +p<0.05, ++p<0.01 Bonferroni corrected for multiple comparisons). BDI = Beck's depression inventory; DMN = default – mode network; mPFC = medial prefrontal cortex; NMI = normalized mutual information.

	beta	t - score	P - value
Forward stepwise			
k_D	-0.39	-4.51	<0.0001
Duration	0.20	1.91	0.061
Degree right TH	0.21	1.74	0.087
Degree left S2	-0.17	-1.64	0.11
Backward stepwise			
k_D	-0.50	-4.40	<0.0001

Supplementary Table 8. Multiple regression of chronic pain intensity with demographic, clinical and brain network parameters. Supplementary Table shows the regression summary for the forward (R^2 = 0.38, $F_{(4,54)}$ = 8.17, p < 0.0001) and backward (R^2 = 0.25, $F_{(1,55)}$ = 19.33, p < 0.0001) stepwise multiple regression analysis of pain intensity(normalized between 0 – 1) with all brain, demographic and clinical parameters list i**n Supplementary Table 7.** All CBP (n = 25), CRPS (n = 22) and OA (n = 20) patients were included in the analysis. Pain intensity showed significant association only with k_D in both models.

Subject	Volumes	TR (seconds)	Slices	Age (years)	Gender	Pain duration (weeks)	BDI (visit 1)	BDI (visit 2)	BDI (visit 3)	VAS (visit 1)	VAS (visit 2)	VAS (visit 3)
SBP01	244	2.5	40	48	F	7	1	2	1	6.1	5.4	5.9
SBP02	244	2.5	40	43	M	9	0	0	1	7.3	6.5	6.1
SBP03	244	2.5	40	57	F	12	2	2	1	8.8	3.5	6.2
SBP04	244	2.5	40	56	M	14	0	1	0	7.8	7.0	8.1
SBP05	244	2.5	40	49	M	8	0	0	1	8.0	3.4	5.0
SBP06	244	2.5	40	56	M	12	0	0	0	2.3	3.9	6.1
SBP07	244	2.5	40	49	F	16	6	5	3	7.7	8.2	8.7
SBP08	244	2.5	40	31	M	10	0	2	3	6.4	7.9	7.2
SBP09	244	2.5	40	44	M	8	1	1	0	6.1	7.3	7.0
SBP10	244	2.5	40	47	F	12	2	1	0	7.6	8.6	8.6
SBP11	244	2.5	40	51	F	12	0	0	1	3.6	4.9	2.5
SBP12	244	2.5	40	33	F	11	0	1	0	7.6	6.9	6.1

Supplementary Table 9. Demographics, scan parameters, and pain characteristics of SBP subjects studied over one year. Healthy controls matched for age and gender were selected from the healthy subject group (see Supplementary Table 2). SBP patients showed no change in reported back pain intensity ($F_{(2,22)}$ = 0.44, p = 0.65, repeated measure ANOVA) and BDI ($F_{(2,22)}$ = 0.49, p = 0.61, repeated measure ANOVA) across the 3 visits.

Subject	Volumes	TR (seconds)	Slices	Age (years)	Gender	BDI	Pain duration (years)	VAS
CBP01	305	2.5	40	54	F	6	8	7.5
CBP02	305	2.5	40	50	M	4	30	6.9
CBP03	305	2.5	40	54	M	4	30	6.2
CBP04	305	2.5	40	62	M	3	25	7.4
CBP05	305	2.5	40	52	M	6	5	5.7
CBP06	305	2.5	40	54	M	4	30	6.2
CBP07	305	2.5	40	58	M	9	10	5.4
CBP08	244	2.5	40	45	M	8	10	8.7
CBP09	244	2.5	40	44	M	0	16	9.4
CBP10	244	2.5	40	48	M	3	12	3.8
CBP11	244	2.5	40	49	M	17	7	6.5
CBP12	244	2.5	40	39	M	5	7	4.4
CBP13	244	2.5	40	51	F	5	6	5.4
CBP14	244	2.5	40	51	M	20	8	6.5
CBP15	244	2.5	40	55	F	2	20	6.9

Supplementary Table 10. Demographics, scan parameters and pain characteristics of CBP subjects used in the validation analysis. BDI is Beck's Depression Index, which is higher than healthy subjects, but all CBP would be classified as moderate or low depression. VAS is visual analog scale. It is used to determine intensity of back pain (0-10 scale).

Subject	Volumes	TR (seconds)	Slices	Age (years)	Gender	BDI	Pain duration (years)	VAS	WOMAC (pain)
OA01	300	2.5	40	60	M	5	4	3.0	3
OA02	300	2.5	40	54	F		6	7.0	11
OA03	300	2.5	40	59	F	2	8	5.0	9
OA04	300	2.5	40	50	M	1	35	7.0	5
OA05	300	2.5	40	61	F	3	23	3.0	8
OA06	300	2.5	40	45	F	0	11	10.0	15
OA07	300	2.5	40	58	F	2	15	8.0	13
OA08	300	2.5	40	48	F	2	13	7.0	5
OA09	300	2.5	40	60	F	14	4	4.0	12
OA10	300	2.5	40	66	M	14	9	7.0	10
OA11	300	2.5	40	45	F	0	5	7.0	10
OA12	300	2.5	40	59	F	4	3	4.5	9
OA13	300	2.5	40	66	M	11	6	7.0	9
OA14	300	2.5	40	59	M	0	27	6.0	10
OA15	300	2.5	40	49	M	3	7	10.0	11
OA16	300	2.5	40	57	F	0	4	8.0	15
OA17	300	2.5	40	60	F	2	2	7.0	10
OA18	300	2.5	40	49	M	16	30	7.0	14
OA19	300	2.5	40	63	F	11	26	7.0	12
OA20	300	2.5	40	64	M	2	30	5.0	8

Supplementary Table 11. Demographics, scan parameters and pain characteristics of OA subjects used in the validation analysis. BDI = Beck's Depression Index; F = female; M=male; VAS = visual analog scale; WOMAC = Western Ontario and McMaster Universities Osteoarthritis Index. Three of these subjects have high depression.

Analysis	Groups	Figure	**Motion (mm)** Mean (s.e.m)	**Correlation (motion- k_D)** r-value (p-value)	Statistics
Chronic pain Patients	CBP	Fig 1d	0.73(0.05)	-0.13(0.64)	$F_{(3,136)}$ = 1.54 p = 0.19 ANOVA
	CRPS	Fig 1d	0.53(0.06)	0.24(0.26)	
	OA	Fig 1d	0.71(0.05)	-0.10(0.69)	
	Healthy	Fig 1d	0.54(0.07)	0.08(0.19)	
Longitudinal SBP	SBP (visit 1)	Fig 6b	0.67(0.06)	0.24(0.44)	$F_{(2,22)}$ = 0.63 p = 0.70, repeated measure ANOVA
	SBP (visit 2)	Fig 6b	0.71(0.07)	0.11(0.72)	
	SBP (visit 3)	Fig 6b	0.60(0.05)	-0.23(0.44)	
	Healthy (visit 1)	Fig 6b	0.68(0.08)	-0.13(0.68)	
	Healthy (visit 2)	Fig 6b	0.67(0.05)	0.05(0.84)	
	Healthy (visit 3)	Fig 6b	0.71(0.09)	0.23(0.44)	
Longitudinal CBP	CBP (visit 1)	Fig S8	0.49(0.09)	0.22 (0.58)	(F(1,14) = 0.98 p = 0.52 repeated measure ANOVA
	CBP (visit 2)	Fig S8	0.53(0.11)	0.60 (0.12)	
	Healthy (visit 1)	Fig S8	0.48(0.06)	-0.12 (0.68)	
	Healthy (visit 2)	Fig S8	0.52(0.09)	0.06 (0.83)	
Validation	CBP	Fig S9	0.57(0.06)	-0.12(0.60)	
	OA	Fig S9	0.60(0.07)	0.01(0.99)	

Supplementary Table 12. No relationship observed between head movement and rank order disruption index. Individual subject head motion was estimated from the translational and rotational parameters obtained by a rigid body transformation of functional volumes relative to its position mid-way through the scan using the MCFLIRT program, part of the FSL software package. Additionally, head motion time courses were regressed out as covariates of no interest during the preprocessing step (see methods for details). Supplementary Table shows the mean ± s.e.m for the patient groups and their corresponding healthy subjects used in the study. In general, absolute head displacements were smaller than 1 mm (smaller than the voxel size) for all functional scans. Relation between k_D and head motion was assessed using a Pearson correlation analysis. There was no significant association between head motion and k_D for all groups examined. Differences in head motion between the patient groups and corresponding healthy controls were computed using the ANOVA or repeated measure ANOVA. There were no significant differences between groups, although in some groups there was a trend.

Analysis	Figure	**Correlation (BDI - k_D)** r -value (p-value)
CBP	Fig 1d	-0.07 (0.78)
OA	Fig 1d	0.14 (0.56)
CRPS	Fig 1d	0.19 (0.57)
SBP (visit 1)	Fig 6b	-0.27 (0.39)
SBP (visit 2)	Fig 6b	-0.32 (0.31)
SBP (visit 3)	Fig 6b	-0.28 (0.35)
CBP (visit 1)	Fig S8	-0.35 (0.32)
CBP (visit 2)	Fig S8	-0.25 (0.54)
CBP (validation)	Fig S9	0.14 (061)
OA (validation)	Fig S9	-0.08 (0.93)

Supplementary Table 13. No relationship observed between depression and rank order disruption index. Individual subject relationship between k_D and depression as assessed by BDI was computed using a Pearson correlation analysis. There was no significant association between BDI and k_D for all patient groups examined.

Supplementary references

1 Yarkoni, T., Poldrack, R. A., Nichols, T. E., Van Essen, D. C. & Wager, T. D. Large-scale automated synthesis of human functional neuroimaging data. *Nat Methods* **8**, 665-670, doi:10.1038/nmeth.1635

2 Power, J. D. *et al.* Methods to detect, characterize, and remove motion artifact in resting state fMRI. *Neuroimage* **84**, 320-341, doi:10.1016/j.neuroimage.2013.08.048 (2014).
